# Targeted genome editing restores auditory function in adult mice with progressive hearing loss caused by a human microRNA mutation

**DOI:** 10.1126/scitranslmed.adn0689

**Published:** 2024-07-10

**Authors:** Wenliang Zhu, Wan Du, Arun Prabhu Rameshbabu, Ariel Miura Armstrong, Stewart Silver, Yehree Kim, Wei Wei, Yilai Shu, Xuezhong Liu, Morag A. Lewis, Karen P. Steel, Zheng-Yi Chen

**Affiliations:** 1 Department of Otolaryngology-Head and Neck Surgery, Graduate Program in Speech and Hearing Bioscience and Technology and Program in Neuroscience, Harvard Medical School, Boston, MA 02115, USA; 2 Eaton-Peabody laboratory, Massachusetts Eye and Ear, Boston, MA 02114, USA; 3 ENT Institute and Otorhinolaryngology Department of Eye & ENT hospital, State Key Laboratory of Medical Neurobiology and MOE Frontiers Center for Brain Science, Fudan University, Shanghai 200031, China; 4 Institutes of Biomedical Science, Fudan University, Shanghai 200032, China; 5 NHC Key Laboratory of Hearing Medicine, Fudan University, Shanghai 200031, China; 6 Department of Otolaryngology, University of Miami School of Medicine, Miami, FL 33136, USA; 7 Wolfson Sensory, Pain and Regeneration Centre, King’s College London, London WC2R 2LS, UK

## Abstract

Mutations in *microRNA-96* (*MIR96*) cause autosomal dominant deafness-50 (DFNA50), a form of delayed-onset hearing loss. Genome editing has shown efficacy in hearing recovery through intervention in neonatal mice, yet editing in the adult inner ear is necessary for clinical applications, which has not been done. Here, we developed a genome editing therapy for the *MIR96* mutation 14C>A by screening different CRISPR systems and optimizing Cas9 expression and the sgRNA scaffold for efficient and specific mutation editing. AAV delivery of the KKH variant of *Staphylococcus aureus* Cas9 (SaCas9-KKH) and sgRNA to the cochleae of presymptomatic (3-week-old) and symptomatic (6-week-old) adult *Mir96*^*14C>A/+*^ mutant mice improved hearing long term, with efficacy increased by injection at a younger age. Adult inner ear delivery resulted in transient Cas9 expression without evidence of AAV genomic integration, indicating the good safety profile of our in vivo genome editing strategy. We developed a dual-AAV system, including an AAV-sgmiR96-master carrying sgRNAs against all known human *MIR96* mutations. Because mouse and human *MIR96* sequences share 100% homology, our approach and sgRNA selection for efficient and specific hair cell editing for long-term hearing recovery lay the foundation for the development of treatment for patients with DFNA50 caused by *MIR96* mutations.

## Introduction

Hearing loss is a multifactorial condition affecting many people world-wide (World Health Organization, https://who.int/news-room/fact-sheets/detail/deafness-and-hearing-loss). Single genetic mutations causing hearing loss account for more than 50% of all congenital sensorineural hearing loss (1), yet no approved pharmaceutical drug or biological treatment is available to slow down or reverse genetic deafness (2–4). Among these deafness genes, microRNAs (miRNAs), which are short [about 20 to 24 nucleotides (nt)] endogenous noncoding RNAs that play a crucial role in the regulation of the expression of protein-coding genes, are considered important factors in the development of the inner ear and are required for normal hearing (5, 6). Specifically, mutations in *MIR96* (*microRNA-96*; *miR-96*) have been identified as causative factors for nonsyndromic progressive hearing loss DFNA50 (autosomal dominant deafness-50) in humans and mice (7–11). A mutation in the seed region of the human *MIR96* is the first example of point mutations in the mature sequence of a miRNA with an etiopathogenic role in a human Mendelian disease (6, 11, 12).

*MIR96* is specifically expressed in sensory cells of the inner ear (13) and plays a critical role in cochlear development and the maintenance of hearing by reducing expression of the mRNAs of multiple target genes (8, 14–16). Point mutations in the seed region of *MIR96* lead to progressive hearing loss with dominant inheritance patterns in human families (10, 11). Mice carrying an *N*-ethyl-*N*-nitrosourea (ENU)–induced mutation, diminuendo (Dmdo), exhibit the phenotype of progressive hearing loss (17). Homozygous Dmdo mice (*Mir96*^*Dmdo/Dmdo*^) and homozygous null mice (knockout of both *Mir96* and *Mir183, Mir183/96*^^*dko/dko*^^) are completely deaf, displaying abnormal hair cell stereocilia bundles and reduction in hair cell numbers in early development, demonstrating the requirement for *Mir96* in normal hair cell development (8, 18). In contrast, heterozygous *Mir96* null mice (*Mir183/96*^*dko/+*^) maintain normal hearing, whereas heterozygous Dmdo mice (*Mir96*^*Dmdo/+*^) develop early-onset nonsyndromic progressive hearing loss, suggesting that the hearing loss phenotype arises from the gain of new target genes rather than loss of function (18). In humans, heterozygous *MIR96* mutations also result in late-onset nonsyndromic progressive hearing loss, which strongly suggests a similar gain of function due to the mutations. Delayed-onset progressive hearing loss offers a window of opportunity to test the intervention for hearing rescue in the mouse models with the goal of human applications.

In addition to its dominant inheritance pattern, overexpression of *Mir96* has been found to cause cochlear defects leading to hearing loss, rendering traditional gene therapy by ectopic *Mir96* expression unsuitable (9). However, genome editing technologies that target the dominant *MIR96* mutations hold great promise in treating DFNA50. In vivo Cas9 nuclease delivery, by Lipofectamine ribonucleoprotein (RNP) delivery (19, 20) or adeno-associated virus (AAV)–mediated delivery, has demonstrated effectiveness in disrupting dominant alleles in multiple hearing loss disease mouse models (19–26). Inner ear base editing has been used to repair a *Tmc1* dominant mutation in mice (27). In all the editing studies of genetic hearing loss, the interventions were done in neonatal mice with immature cochleae (28). In contrast, newborn human inner ears are fully developed (29). The mouse cochlea undergoes developmental changes from the neonatal to adult stages, including changes in size, structure, and function (28). This distinction highlights the need to evaluate the efficacy, safety, and time window of genome editing treatment in the mature cochlea to establish the feasibility of intervention for potential clinical applications.

Here, we performed editing by nonhomologous DNA end joining (NHEJ) to target a dominant *Mir96* mutation (*Mir96*^*tm3.1Wtsi*^, referred to as *Mir96*^*14C>A*^ in this report; equivalent of human rs587776523, g.7:129414596G>T in GRCh38) by AAV-mediated delivery in young adult mice with hearing loss. We showed that AAV-mediated delivery of the editing complex was well tolerated without impairing normal hearing. We screened different editors and identified one for optimization, resulting in efficient and specific editing in vitro and in vivo. We tested interventions before and after the onset of hearing loss and established a time window when the treatment results in sustained hearing preservation. To improve the safety profile of the editing strategy, we established the dose range without detectable AAV integrations and showed the transient SaCas9-KKH expression that ended 12 weeks after injection. Because human and mouse *Mir96* share 100% sequences in the seed region, we developed a dual-AAV system capable of targeting multiple *MIR96* mutations, providing evidence for its potential generalization and broad utility. In conclusion, our study establishes the feasibility of in vivo genome editing as a viable approach for treating hearing loss caused by genetic mutations in adult animals.

## Results

### A human *microRNA-96* mutation *Mir96*^*14C>A*^ causes hearing loss

**Fig. 1:**
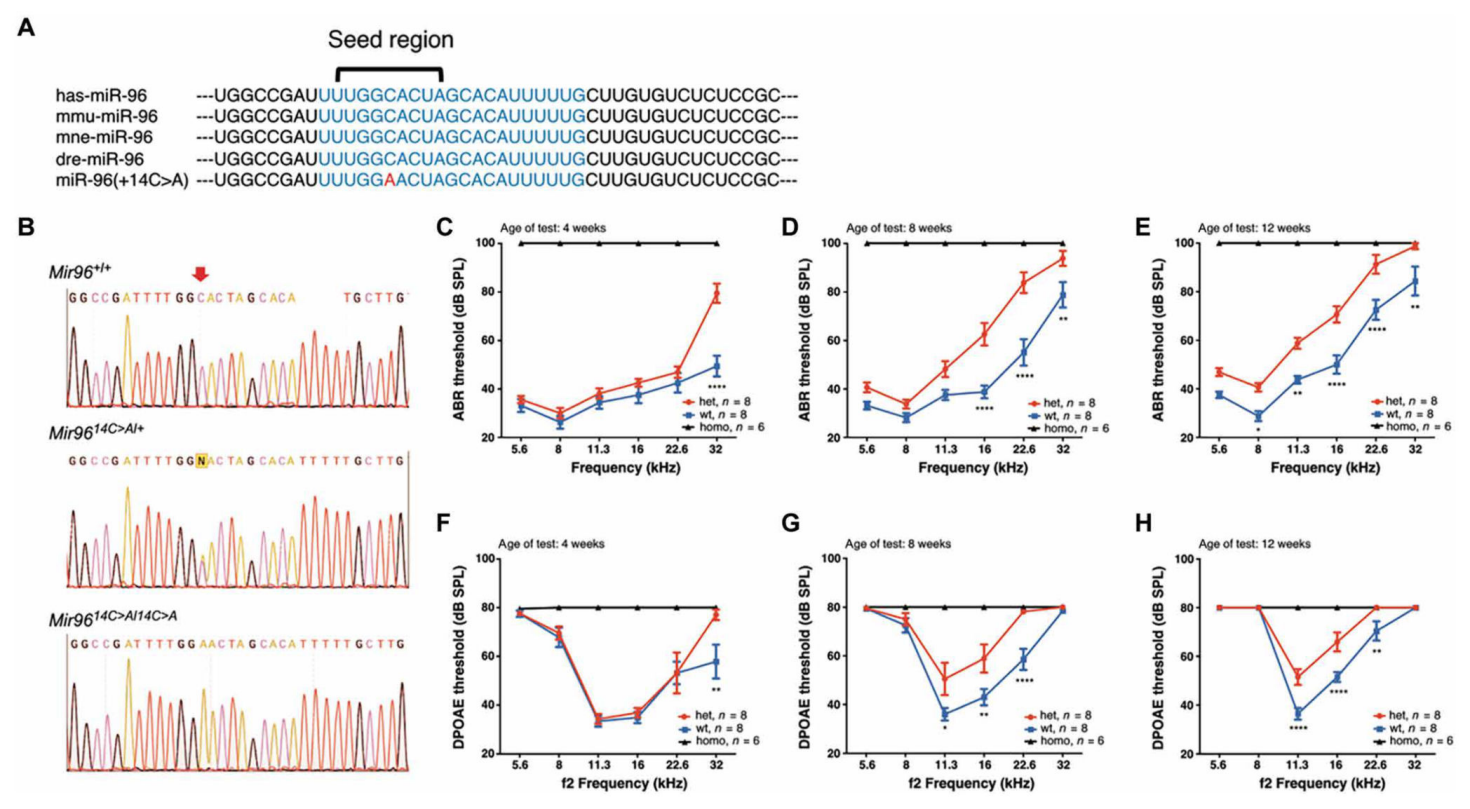
*Mir96*^*14C*>*A*^ mutant mice present early-onset progressive hearing loss. (**A**) *Mir96* sequence in human (has-), mouse (mmu-), macaque (mne-), zebrafish (dre-), and 14C>A mutation. The blue text region shows 100% conservation. The mutated nucleotide in *Mir96*^*14C*>*A*^ is displayed in red. (**B**) Representative Sanger sequencing results showed the *Mir96* mutation locus in WT mice, *Mir96*^*14C*>*A/*+^, *Mir96*^*14C*>*A/14C*>*A*^. The red arrow indicates the mutated nucleotide. (**C** to **E**) ABR thresholds in *Mir96*^*14C*>*A/*+^ (het) mice (red) compared with WT mice (blue) and *Mir96*^*14C*>*A/14C*>*A*^ (homo) mice (black) at 4 weeks (C), 8 weeks (D), and 12 weeks (E), respectively. (**F** to **H**) DPOAE thresholds in *Mir96*^*14C*>*A/*+^ ears (red) compared with WT ears (blue) and *Mir96*^*14C*>*A/14C*>*A*^ ears (black) at 4 weeks (F), 8 weeks (G), and 12 weeks (H), respectively. Values and error bars reflect mean ± SEM. Statistical analyses were performed by two-way ANOVA with Bonferroni correction for multiple comparisons: *P < 0.05, **P < 0.01, ***P < 0.001, and ****P < 0.0001. WT refers to WT C57BL/6N mice.

MiR-96 is highly expressed in mammalian sensory cells and functions as a master regulator controlling expression of many genes (8, 17). *Mir96* is super conserved across vertebrates, from zebrafish to humans, with the nucleotides within the *Mir96* seed region showing 100% identity in DNA sequence (Fig. 1A).

Two mutations in the seed region, +13G>A (rs587776522) and +14C>A (rs587776523), have been identified as the cause of autosomal dominant nonsyndromic progressive hearing loss DFNA50 (11). Our mouse model carries the *Mir96*^*14C>A*^ mutation, which substitutes the 14th nucleotide of cytosine in the conserved seed region with adenine (30) (Fig. 1, A and B). To evaluate how hearing is affected by the mutation, we performed auditory brainstem response (ABR) (Fig. 1, C to E) and distortion product otoacoustic emission (DPOAE) (Fig. 1, F to H) tests on *Mir96*^*14C>A*^ mice. *Mir96*^*14C>A/14C>A*^ mice exhibited complete hearing loss across all frequencies and age groups by ABR and DPOAE at 4 weeks of age. In *Mir96*^*14C>A/+*^ mice at 4 weeks of age, we observed a 30-dB elevation in ABR (Fig. 1C) and a 19-dB elevation in DPOAE (Fig. 1F) at the frequency of 32 kHz but no significant (*P* > 0.05) changes in ABR/DPOAE thresholds at other frequencies compared to wild-type (WT) ears, indicating the onset of hearing loss starting at 4 weeks at 32 kHz. By 8 weeks, the ABR thresholds were significantly (*P* < 0.01) elevated from 16 to 32 kHz, with an average increase of 20 dB in *Mir96*^*14C>A/+*^ ears compared with WT ears. Similarly, DPOAE thresholds were significantly (*P* < 0.05) elevated by an average of 16 dB from 11.3- to 22.6-kHz frequencies in *Mir96*^*14C>A/+*^ ears compared with WT ears (Fig. 1G). At 12 weeks, the hearing loss became more severe, because ABR thresholds were significantly (*P* < 0.01) elevated across all frequencies when compared with WT ears, with an average elevation of 15 dB. In addition, DPOAE thresholds were significantly (*P* < 0.01) elevated by 15 dB on average compared with WT ears, particularly at 11.3 and 16 kHz (Fig. 1H). These results showed that *Mir96*^*14C>A/+*^ mice exhibit hearing loss starting at 4 weeks at the high frequency of 32 kHz, which becomes progressive with further elevation of ABR/DPOAE thresholds at 8 and 12 weeks across other frequencies.

### Screening of genome editing systems in mouse and human cells

**Fig. 2:**
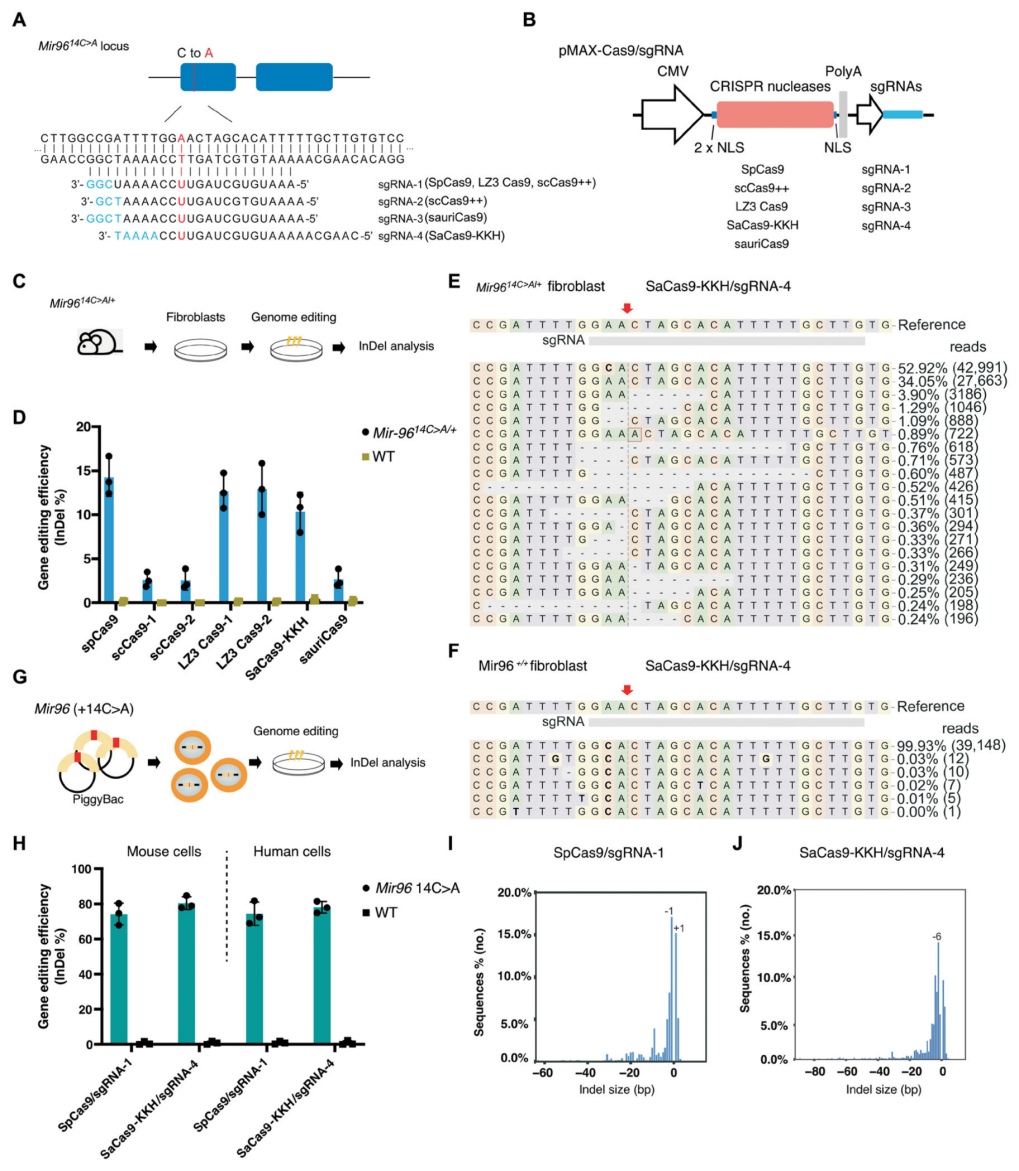
*Mir96 14C*>*A* allele-specific genome editing using different CRISPR systems in mouse and human cells. (**A**) Illustration of the *Mir96* + 14 C>A mutation locus and sgRNA sequences. The mutated nucleotide in the *Mir96*^*14C*>*A*^ allele is displayed in red. The protospacer adjacent motif (PAM) nucleotides are displayed in blue. (**B**) Schematic overview of plasmid constructions for different CRISPR systems. (**C**) Illustration of primary fibroblast isolation from *Mir96*^*14C*>*A/*+^ mice and subsequent genome editing and InDel analysis. (**D**) Bar chart showing InDel frequencies in *Mir96*^*14C*>*A/*+^ and WT primary fibroblasts after genome editing using different Cas9/sgRNA combinations. *n* = 3 per treatment condition. Values and error bars reflect mean ± SD. (**E** and **F**) Representative NGS results from SaCas9-KKH/sgRNA-4 edited *Mir96*^*14C*>*A/*+^ and WT primary fibroblasts. The red arrow indicates the double-stranded DNA cutting site. Reference sequence is the mutant allele. (**G**) Overview of human and mouse *MIR96* + 14 C>A cell line generation; DNA fragment containing the *MIR96* + 14 C>A mutation was integrated into the genome of different cell lines using PiggyBac transposons technology. (**H**) Bar chart showing the editing efficiency of *Mir96* mutation locus and WT locus using SpCas9/sgRNA-1 and SaCas9-KKH/sgRNA-4 (*n* = 3). Each dot represents a unique sequencing reaction. Values and error bars reflect mean ± SD. (**I** and **J**) InDel profiles from SpCas9/sgRNA-1 and SaCas9-KKH/sgRNA-4 edited *Mir96* + 14C>A HEK-293T cells. Negative numbers represent deletions, and positive numbers represent insertions.

To specifically disrupt the *Mir96*^*14C>A*^ allele, single guide RNAs (sgRNAs) were designed to target the allele by each of the five types of CRISPR nucleases: SpCas9 (31), scCas9++ (32), SaCas9-KKH (33), sauriCas9 (34), and LZ3 Cas9 (35) (Fig. 2A). All Cas9/sgRNA combinations were in the same pMAX-Cas9/sgRNA backbone with three nuclear localization signals (NLSs) to ensure consistency in plasmid structure (Fig. 2B and fig. S1A). We delivered the CRISPR systems into *Mir96*^*14C>A/+*^ fibroblasts by nucleofection and analyzed insertion and deletion (InDel) frequency by next-generation sequencing (NGS). SpCas9/sgRNA-1 exhibited the highest editing efficiency (14.3 ± 2.3%) (Fig. 2, C and D). LZ3 Cas9/sgRNA-1 and SaCas9-KKH/sgRNA-4 also showed comparable editing efficiency (12.7 ± 2.0% and 10.1 ± 2.1%), whereas scCas9++/sgRNA-1, scCas9++/sgRNA-2, and sauriCas9/sgRNA-4 displayed modest InDel frequency (Fig. 2, C and D). SpCas9/sgRNA-1 and SaCas9-KKH/sgRNA-4 exhibited high specificity in targeting the *Mir96*^*14C>A*^ allele; NGS analysis showed that all the InDels occurred exclusively at the mutant allele and were undetectable in *Mir96*^+/+^ cells (less than 0.03%) (Fig. 2, D to F, and fig. S1B). We compared the editing efficiency of Cas9/sgRNA RNP and plasmids in primary fibroblasts of *Mir96*^*14C>A/+*^ mice. RNP nucleofection yielded the highest editing efficiency, whereas Lipofectamine-mediated RNP delivery was less efficient (fig. S1C). The AAV systems with SpCas9/sgRNA1 dual-AAV plasmids (7.3 ± 1.5%) and SaCas9-KKH/sgRNA4 (10.4 ± 1.8%) single-AAV plasmid exhibited editing frequencies comparable to the pMAX-Cas9 plasmid transfection (14.3 ± 2.3%) (fig. S1C).

Because of 100% homology in the *Mir96* seed region across species, the sgRNA design targeting the *Mir96*^*14C>A*^ allele in mice can be used in the human *MIR96* with the same mutation. To validate the editing systems in human cells, we generated human and mouse cell lines carrying the *Mir96*^*14C>A*^ mutation using the PiggyBac transposon system (Fig. 2G). In cells transfected with SpCas9/sgRNA-1 and SaCas9-KKH/sgRNA-4, NGS analysis showed a high percentage of InDel reads in both human and mouse *Mir96* + 14C>A cell lines, where negligible InDels were observed in the *Mir96* WT cell lines (Fig. 2H). The InDel profile showed various types of InDels for SpCas9/sgRNA-1 and SaCas9-KKH/sgRNA-4, with single-nucleotide deletions (−1 and +1) being the most common type for SpCas9/sgRNA-1 and a 6-nt deletion being the most common for SaCas9-KKH/sgRNA-4 (Fig. 2, I and J).

In addition to disrupting the mutated allele with CRISPR nucleases, we also attempted to correct the *Mir96*^*14C>A*^ mutation using prime editing (36–38) (fig. S2A). We designed a pegRNA located upstream of the mutate nucleotide, containing pegRNA-extensions with 13–base pair (bp) primer binding sites (PBSs) and 16-bp RT-templates harboring the corrected sequence (fig. S2B). After prime editing, NGS analysis showed that the mutation was corrected in *Mir96*^*14C>A*^ cells with an efficiency of 2.8 ± 1.4% (fig. S2, C and D). However, because of the modest efficiency of prime editing at this locus and the lack of an efficient in vivo delivery system for the inner ear, CRISPR nuclease-mediated knockout of the mutated allele was selected for treating hearing loss caused by the *Mir96* mutation.

### Optimization of the CRISPR/sgRNA delivery system for cochlea in adult mice

We chose AAV serotype 2 (AAV2) for delivering Cas9/sgRNA to hair cells, because it has demonstrated high efficiency and specificity in transducing mature inner hair cells (IHCs) and outer hair cells (OHCs) compared with other AAV serotypes (39, 40). To further assess the delivery efficiency of AAV2, we initially delivered AAV2 carrying a green fluorescent protein (GFP) cassette into the adult cochlea through round window membrane and canal fenestration (RWM + CF) injection in 18-week-old mice (fig. S3A). The animals that underwent RWM + CF gene delivery presented no hearing loss (fig. S3, B and C), suggesting that AAV2 was well tolerated without impairing normal hearing. Four weeks after injection, robust GFP expression was observed in nearly all IHCs throughout the cochlear turns and in a majority of OHCs with an apex-to-base gradient (fig. S3, D to F). Most GFP^+^ cells were hair cells, with only limited GFP^+^ cells observed in other cell types, indicating that AAV2 specifically and efficiently targets auditory hair cells in adult mice.

**Fig. 3:**
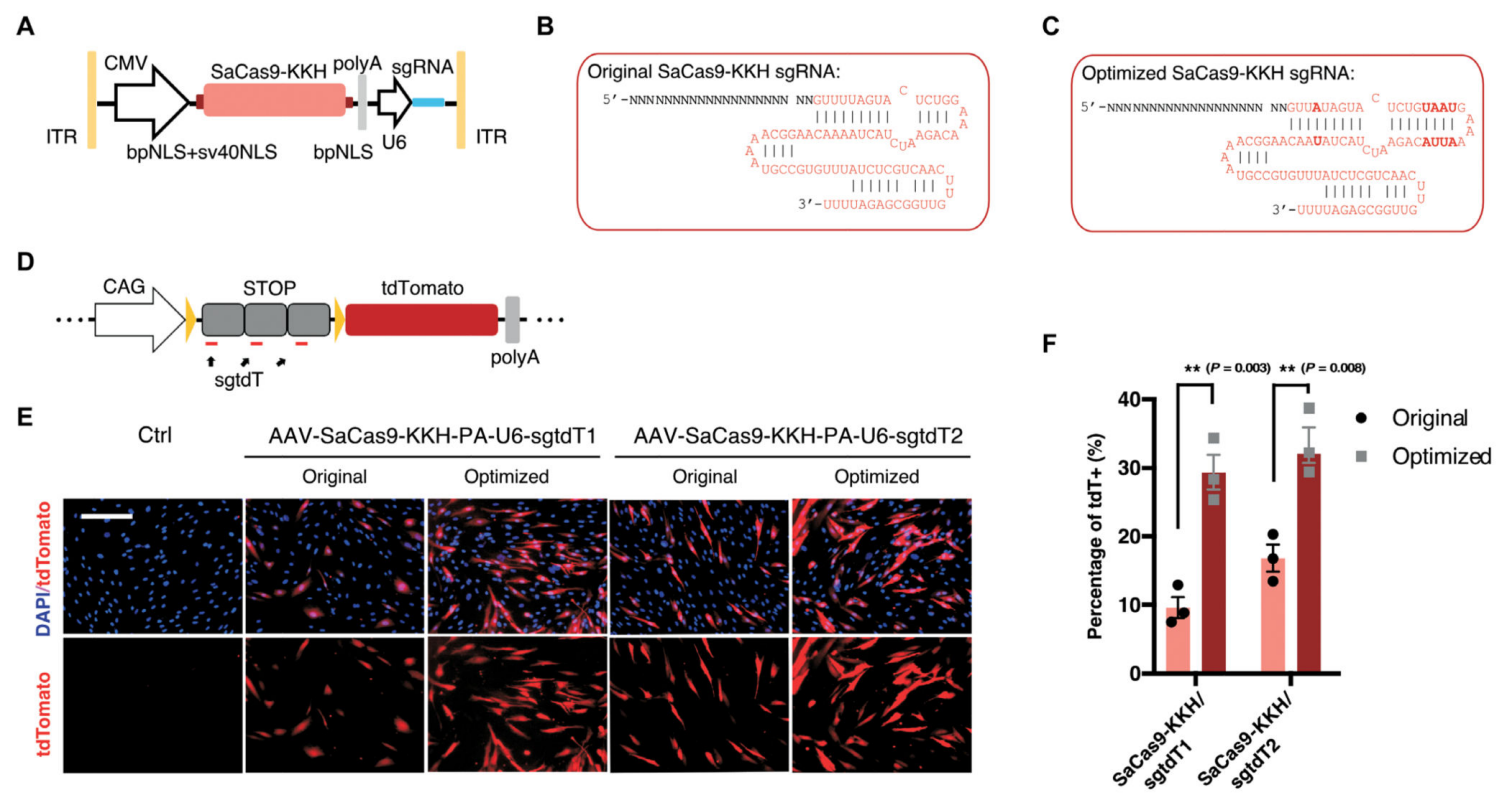
Optimization of CRISPR constructs for inner ear delivery. (**A**) The design of the optimized AAV structure of SaCas9-KKH sgRNA vector with multiple NLS sites. (**B** and **C**) Sequence of unmodified and optimized SaCas9-KKH sgRNA, with sequence changes in bold. (**D**) Schematic view of the tdTomato reporter structure in the primary fibroblasts. (**E**) Representative fluorescence images of primary fibroblasts after editing by unmodified and optimized SaCas9-KKH/sgRNA systems. tdTomato^+^ cells are edited. Three technical replicates. (**F**) Bar chart showing the editing efficiency by the quantification of tdTomato^+^ cells after editing. Values and error bars reflect mean ± SD. Each dot represents one independent experiment.

To maximize editing efficiency in vivo to improve hearing rescue, we modified the AAV Cas9/sgRNA construct by incorporating a bipartite nuclear localization signal (bpNLS), an sv40 NLS at the N termini, and another bpNLS at the C termini (Fig. 3A) to enhance nuclear import (41, 42). The SaCas9-KKH sgRNAs were optimized by using an 84-nt sgRNA (crRNA-tracrRNA duplex extension), which exhibits higher activity than the canonical full-length sgRNA (33). In addition, we introduced a third A-U flip in the stem-loop to remove a putative RNA Pol III terminator sequence (four consecutive U’s) to further enhance expression under the U6 promoter (43) (Fig. 3, B and C). The optimized construct was tested in the primary fibroblasts derived from Ai14 mice, which contain a “CAG-STOP-tdTomato” cassette (44) (Fig. 3D). In the cells transfected with the optimized SaCas9-KKH/sgRNA constructs, an increase in editing efficiency of approximately 3.05- and 1.98-fold was detected for SaCas9-KKH/sgtdT-1 and SaCas9-KKH/sgtdT-2, respectively (Fig. 3, E and F). The editing efficiency was also evaluated in human embryonic kidney (HEK)–GFP cells using SaCas9-KKH/sgGFP targeting GFP, and the ratio of GFP^–^ cells showed significantly (*P* < 0.01) higher efficiency when using the optimized AAV constructions compared with the original design (fig. S4, A and B). The optimized AAV plasmid design and gRNA modifications were used for the production of AAV-SaCas9-KKH/sgRNA-4 for the in vivo editing study.

### In vivo genome editing of the *Mir96*^*14C>A*^ allele in mature cochleae

**Fig. 4:**
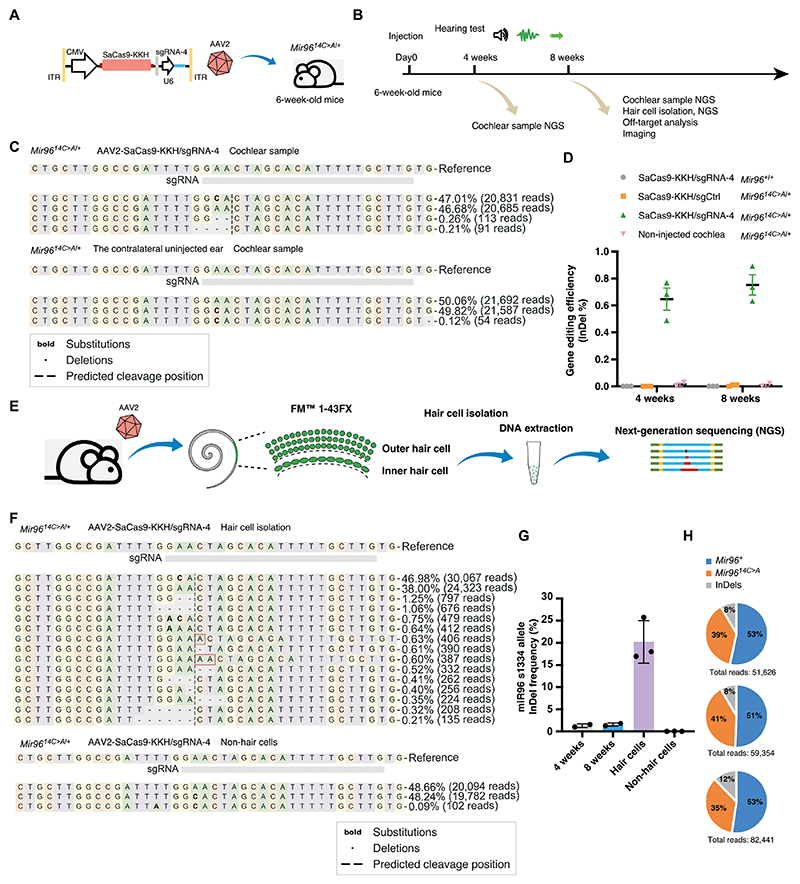
AAV2-CRISPR mediated targeted genome editing at the *Mir96*^*14C*>*A*^ locus in hair cells of *Mir96*^*14C*>*A/*+^ mice. (**A**) Schematic view of the AAV2-CMV-SaCas9-KKH-sgRNA4 design. (**B**) Timeline of in vivo studies. (**C**) Representative NGS results of cochlea samples from AAV2-SaCas9-KKH-sgRNA-4–injected and the contralateral uninjected ears of *Mir96*^*14C*>*A/*+^ mice. Reference sequence is the mutant allele. (**D**) Quantification of InDel frequency from AAV2-SaCas9-KKH-sgRNA-4–injected, AAV2-SaCas9-KKH-sgCtrl–injected, and uninjected ears from *Mir96*^*14C*>*A/*+^ mice, as well as AAV2-SaCas9-KKH-sgRNA-4–injected ears from WT mice (*n* = 9). Cochleae were collected 4 and 8 weeks after AAV injection. Each dot represents a unique sequencing reaction from a combination of three cochleae. Values and error bars reflect mean ± SD. (**E**) Schematic overview of the experimental protocol of hair cell isolation, cell lysis, and NGS. (**F**) Representative NGS result of isolated hair cells from AAV2-SaCas9-KKH-sgRNA-4–injected cochlea. Reference sequence is the mutant allele. (**G**) Quantification of *Mir96*^*14C*>*A*^ allele-specific InDel frequency from NGS of hair cell and cochlea samples after AAV2-SaCas9-KKH-sgRNA-4 injection (*n* = 3). Each dot represents a unique sequencing reaction from three cochleae combination. Values and error bars reflect mean ± SD. (**H**) Percentage of *Mir96* WT allele reads, 14C>A reads, and InDel-containing reads in the NGS results from AAV2-SaCas9-KKH-sgRNA-4–injected hair cells from three independent experiments.

SaCas9-KKH/sgRNA-4 and SaCas9-KKH/sgCtrl were packaged into AAV2 and delivered to the cochleae of 6-week-old *Mir96*^*14C>A/+*^ mice through RWM + CF injection at a dose of 6 × 10^9^ vg (Fig. 4A). SaCas9-KKH/sgtdT-1 (Fig. 3D) was used as SaCas9-KKH/sgCtrl, because it does not target any endogenous loci in the mouse genome. Cochleae were collected 4 and 8 weeks after injection for NGS (Fig. 4B). NGS of cochlear tissue detected the presence of InDels at the *Mir96* mutant locus in the injected ear at 4 and 8 weeks, but no InDel was detected in the contralateral uninjected ears (Fig. 4, C and D, and fig. S5, A to C). AAV2-SaCas9-KKH-sgRNA-4 specifically edited the *Mir96*^*14C>A*^ allele, because no InDel was observed in AAV2-SaCas9-KKH/sgRNA-4–treated WT mice or AAV2- SaCas9-KKH/sgCtrl–treated *Mir96*^*14C>A/+*^ mice (Fig. 4D and fig. S5, A to C). Eight weeks after injection, the InDel rate was determined to be 0.76 ± 0.13%, slightly higher than the rate observed 1 month after injection (0.63 ± 0.14%) (Fig. 4D). However, the InDel frequency from organ of Corti samples does not accurately reflect the editing efficiency in hair cells, which constitutes less than 5% of cochlear cells.

To obtain a precise measurement of editing efficiency in hair cells, we performed injection and assessed isolated hair cells after labeling hair cells with the FM1-43 uptake assay (45, 46) (fig. S6A). The labeled hair cells were handpicked under an inverted fluorescent microscope and lysed, with DNA extracted for polymerase chain reaction (PCR) and NGS (Fig. 4E). Robust InDel formation was observed in isolated hair cells from injected animals 8 weeks after treatment, with an InDel frequency of 16.3 to 25.73% in the *Mir96*^*14C>A*^ allele and a wider range of InDel types, including −2 bp, −4 bp, +1 bp, and −1 bp (Fig. 4, F and G, and fig. S6, C to E), indicating efficient targeted genome editing in hair cells. From three independent experiments, the ratio of *Mir96* WT allele reads to *Mir96*^*14C>A*^ allele reads was analyzed on the basis of NGS results after editing. In the injected ear, the ratio between the *Mir96*^*14C>A*^ allele that combined unedited with InDel-containing reads and that of *Mir96* WT reads was 47.7%:52.3% (Fig. 4H), similar to that observed in uninjected ears (fig. S6, B and F), indicating no noticeable chromatin lesion, insertion, or large deletions caused by genome editing.

### In vivo genome editing improved short- and long-term hearing preservation

**Fig. 5:**
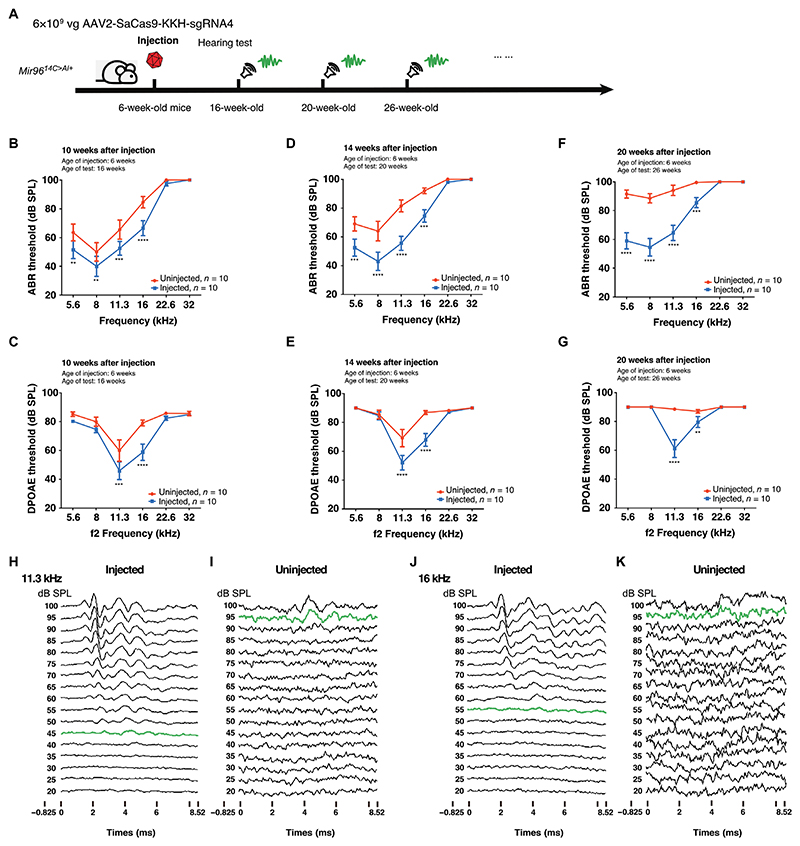
AAV2-SaCas9-KKH-sgRNA-4 delivery into cochleae of 6-week-old *Mir96*^*14C*>*A/*+^ mice promotes sustained rescue of hearing in adulthood. (**A**) Timeline of AAV2-SaCas9-KKH-sgRNA-4 delivery into adult *Mir96*^*14C*>*A/*+^ mouse cochleae and subsequent auditory function assays. (**B** to **G**) Frequency-dependent ABR and DPOAE thresholds in injected (blue) versus uninjected contralateral ears (red) at 16 weeks of age (B and C), 20 weeks of age (D and E), and 26 weeks of age (F and G). *n* = 10. (**H** to **K**) Representative ABR waveforms recorded from an injected (left) and an uninjected ear (right) of a mouse of 20 weeks of age in response to 11.3-kHz (H and I) and 16-kHz auditory stimulation (J and K). Single traces represent responses to different stimulation intensities [20 to 100 decibels (dB)]. The thresholds were determined by the detection of peak 1 (green color traces). Values and error bars reflect mean ± SEM. Statistical analyses were performed by two-way ANOVA with Bonferroni correction for multiple comparisons: *P < 0.05, **P < 0.01, ***P < 0.001, and ****P < 0.0001.

We injected AAV2-SaCas9-KKH-sgRNA-4 into the cochleae of 6-week-old (P40 to P45) *Mir96*^*14C>A/+*^ mice through RWM + CF, with the contralateral uninjected ears serving as controls (Fig. 5A). Ten weeks after the injection, at 16 weeks of age, the injected ears showed overall lower ABR thresholds compared with the uninjected control ears, at frequencies from 5.6 to 16 kHz (Fig. 5B), an indication of better hearing. The ABR threshold reduction ranged from 12 dB at 5.6 kHz to 18 dB at 16 kHz for frequencies below 22.6 kHz, with an average reduction of 13 dB. In addition, lower DPOAE thresholds were observed in the injected ears compared with the control ears at frequencies of 11.3 and 16 kHz, with reductions of 14 to 21 dB, respectively (Fig. 5C).

To assess long-term auditory function after hair cell–targeted genome editing, hearing tests were conducted over an extended period. Fourteen weeks after injection (20 weeks of age), significantly (*P* < 0.001) lower ABR thresholds were detected in injected ears compared to the uninjected control ears at frequencies of 5.6, 8, 11.3, and 16 kHz, with an average reduction of 21 dB in ABR thresholds. At the frequencies of 8 and 11.3 kHz, the reduction was 21 and 26 dB, respectively (Fig. 5D). Significant (*P* < 0.0001) reductions in DPOAE thresholds were observed in the injected ears compared with the uninjected control ears at the frequencies of 11.3 and 16 kHz, with reductions at 15 and 19 dB, respectively (Fig. 5E). Twenty weeks after injection (26 weeks age), more marked improvements in ABR thresholds were detected in the injected ears compared with the uninjected control ears from 5.6 to 16 kHz, with an average reduction of 28 dB in ABR thresholds (Fig. 5F). For the frequencies of 5.6 and 8 kHz, the ABR thresholds were reduced by 33 and 34 dB, respectively. Similarly, markedly lower DPOAE thresholds were detected in injected ears compared with uninjected control ears at frequencies of 11.3 and 16 kHz, with reductions of 27 and 7 dB, respectively (Fig. 5G). Representative ABR waveforms recorded from an AAV2-SaCas9-KKH-sgRNA-4–injected ear and the contralateral uninjected ear 14 weeks after injection using 11.3-kHz (Fig. 5, H and I) and 16-kHz (Fig. 5, J and K) tone bursts at incrementally increasing sound pressure levels (SPLs) from 20 to 100 dB further illustrated the improved hearing preservation. Collectively, these results demonstrate that AAV2-mediated genome editing effectively abolished the expression of the mutant *Mir96*, leading to improved short- and long-term hearing preservation.

Delayed-onset progressive hearing loss caused by *MIR96* mutations offers a window of opportunity for intervention. To determine the optimal time window for genome editing treatment in *Mir96*^*14C>A/+*^ mice, we further performed AAV2-SaCas9-KKH-sgRNA-4 injections in 3-week-old mice in which initial hearing loss was detected only at the high frequency and in 16-week-old mice in which severe hearing loss was detected at all frequencies (Fig. 1, C and F, and fig. S7A). For 3-week-old injection, significantly (*P* < 0.01) lower ABR thresholds were detected 13 weeks after injection in AAV2-SaCas9-KKH-sgRNA-4–treated ears compared with the untreated control ears, with an average ABR threshold reduction of 19 dB from 5.6 to 16 kHz (fig. S7B). A lower ABR threshold of 8 dB was seen at 22.6 kHz, although the reduction was not statistically significant (*P* > 0.5). The average ABR threshold reduction in *Mir96*^*14C>A/+*^ mice injected at 3 weeks of age was greater than that of mice injected at 6 weeks of age, 19 dB versus 13 dB. Significantly (*P* < 0.001) lower DPOAE thresholds were also detected in the AAV2-SaCas9-KKH-sgRNA-4–treated ears compared with the contralateral control ears at frequencies of 11.3 and 16 kHz, with a reduction of 31 dB at 11.3 kHz and 24 dB at 16 kHz. A lower DPOAE threshold of 9 dB was detected at 22.6 kHz; however, it was not statistically different (fig. S7C). Again, the average DPOAE threshold reduction in *Mir96*^*14C>A/+*^ mice injected at 3 weeks old was greater than that in *Mir96*^*14C>A/+*^ mice injected at 6 weeks old, 27 dB versus 18 dB.

In mice injected at 16 weeks of age, no difference in ABR and DPOAE thresholds was detected between AAV2-SaCas9-KKH-sgRNA-4–treated and contralateral control ears 10 weeks later (fig. S7, D and E). These data indicate that genome editing therapy is no longer an effective treatment at later stages of severe hearing loss. Combined with the data from 3-, 6-, and 16-week injections, the results highlight the importance of early intervention for more effective outcomes before substantial hearing loss is initiated.

### In vivo genome editing preserves hair cell survival

**Fig. 6:**
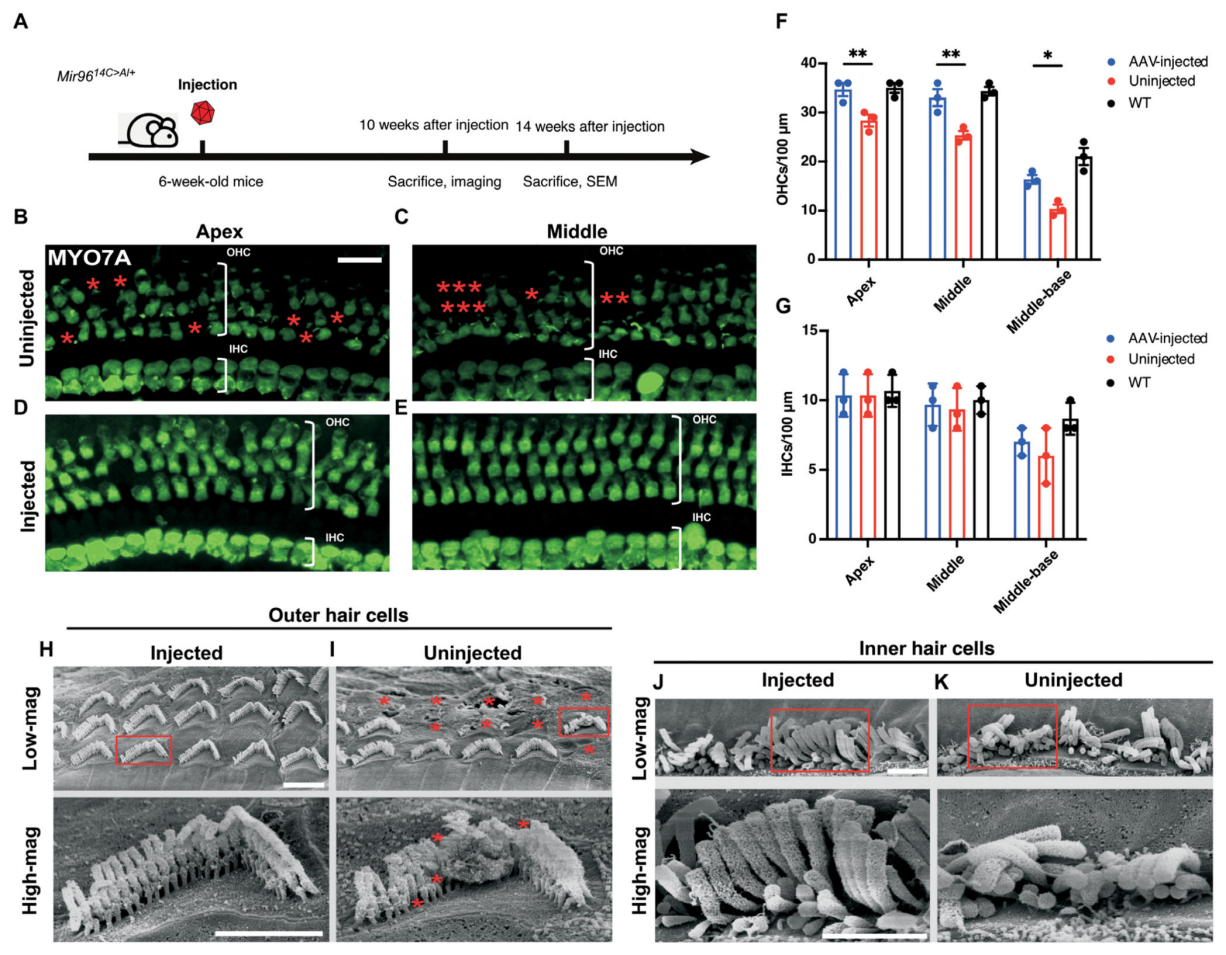
AAV2-SaCas9-KKH-sgRNA-4 delivery into cochleae of adult *Mir96*^*14C*>*A/*+^ mice promotes hair cell survival and maintenance of stereocilia integrity. (**A**) Timeline of AAV2-SaCas9-KKH-sgRNA-4 delivery, confocal analysis, and SEM study of ears harvested at 10 and 14 weeks after injection, respectively. (**B** to **E**) Representative confocal z-stack images of whole-mount cochleae from uninjected (B and C) and AAV2-SaCas9-KKH-sgRNA-4–injected (D and E) *Mir96*^*14C*>*A/*+^ mice. Hair cells were stained for MYO7A (green). Asterisks point to missing hair cells. Scale bar, 20 μm. Experiments were repeated independently in three cochleae. (**F** and **G**) Quantification and comparison of the number of OHCs (F) and IHCs (G) across the cochlear turns from injected and uninjected *Mir96*^*14C*>*A/*+^ mice 10 weeks after injection. Uninjected WT mice were used as a control. Error bar represents SD. *P < 0.05, **P < 0.01, ***P < 0.001, and ****P < 0.0001. (**H** and **I**) SEM images of injected (H) uninjected (I) *Mir96*^*14C*>*A/*+^ OHC bundles at the apical turn. The asterisks indicate the missing stereocilia in an OHC from an uninjected ear. Scale bar, 2 μm. (**J** and **K**) Images of SEM of injected (J) and uninjected (K) *Mir96*^*14C*>*A/*+^ IHC bundles at the apex turn. Scale bar, 2 μm.

To test the effect of editing on HCs in vivo, we injected the AAV2-SaCas9-KKH-sgRNA-4 into the inner ears of 6-week-old *Mir96*^*14C>A/+*^ mice. Cochleae were harvested 10 weeks after the injection for hair cell labeling using an anti-MYO7A antibody and confocal imaging (Fig. 6A). In the uninjected *Mir96*^*14C>A/+*^ inner ears, OHC loss across cochlear turns was observed, with the most severe loss in the basal turn (Fig. 6, B and C, and fig. S8, A and B), whereas only a modest OHC loss was observed in WT animals (fig. S8, C to E). For IHCs, a slight loss was detected in the basal turn only (Fig. 6, F and G, and fig. S8A). In the injected *Mir96*^*14C>A/+*^ ears, improved OHC survival was seen across all frequency regions compared with the uninjected *Mir96*^*14C>A/+*^ mice (Fig. 6, D to F, and fig. S8B), whereas a slight reduction in the IHC number was seen in the base similar to uninjected ears (Fig. 6G and fig. S8, A and B). To further examine hair cell structure, scanning electron microscopy (SEM) was performed to visualize the details of OHC and IHC stereocilia. Cochleae from uninjected and AAV2-SaCas9-KKH-sgRNA-4–injected *Mir96*^*14C>A/+*^ mice were harvested at 14 weeks after injection and imaged by SEM (Fig. 6A). Hair cells from the uninjected *Mir96*^*14C>A/+*^ mice cochleae showed signs of degeneration, including missing stereocilia in the OHCs and disorganized stereocilia in the IHCs. In contrast, the hair cells of injected *Mir96*^*14C>A/+*^ mouse cochlea had organized and well-preserved stereocilia (Fig. 6, H to K). These findings demonstrate that in vivo genome editing by AAV2-SaCas9-KKH-sgRNA-4 rescues hair cells in *Mir96*^*14C>A/+*^ mice by promoting their survival and maintaining the structure of stereocilia.

### Safety assessment of AAV-mediated genome editing treatment in adult mice

**Fig. 7:**
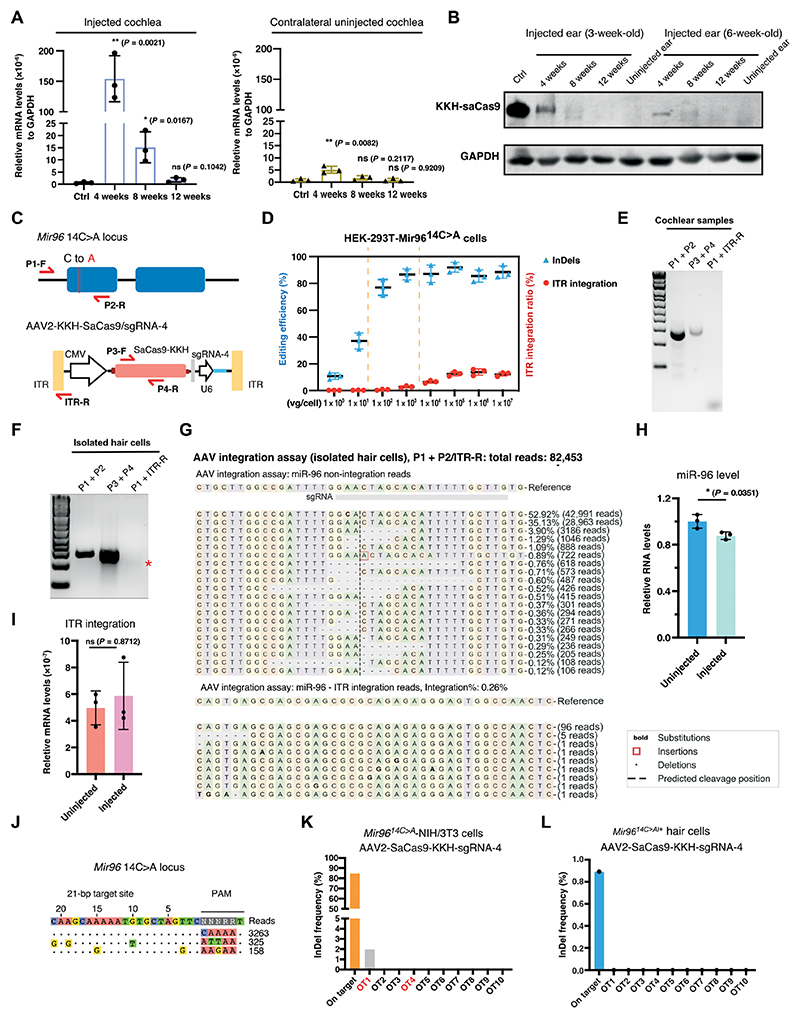
Safety assessment of AAV2-SaCas9-KKH-sgRNA-4 delivery into cochleae of 3- and 6-week-old mice. (**A**) qPCR of SaCas9-KKH expression in the injected cochleae and the contralateral uninjected cochleae in 6-week-old mice. Ctrl refers to the uninjected cochlea. Each dot represents data from a combination of two cochleae (*n* = 6). (**B**) Western blotting of SaCas9-KKH protein in both 3-week-old and 6-week-old injected cochleae and the contralateral uninjected cochleae. (**C**) Primer design for the AAV vector integration assay; the red arrows indicate the location of the primers; P1-F and P2-R were used for amplifying *Mir96* loci in mouse genome; P3-F and P4-R were used for detecting AAV vector; P1-F and ITR-R were used for detecting AAV vector integration at *Mir96* loci. (**D**) Quantification of InDel frequency and AAV vector ITR integration ratio from edited HEK-293T-*Mir96*^*14C*>*A*^ cells. The vertical dashed lines represent the optimal dosage range. (**E** and **F**) Gel image of the PCR products, showing the bands of the *Mir96* locus, AAV vectors, and miR96-ITR integration in edited cochlea samples (E) and isolated hair cells from injected ears (F). Asterisk indicates the putative integration fragment position. (**G**) MiR96-ITR integration reads from NGS of isolated hair cells from injected ears. (**H**) qPCR analysis of *Mir96* in injected and uninjected cochleae. Each dot represents an independent result from two cochleae combined (*n* = 6). (**I**) qPCR analysis of miR96-ITR RNA in injected and uninjected cochleae (*n* = 3). (**J**) CIRCLEseq analysis of SaCas9-KKH-sgRNA-4 in *Mir96*^*14C*>*A*/+^ primary fibroblasts genomic DNA. (**K** and **L**) Quantification of InDel frequency of potential off-target sites in vitro (K) and in vivo (L). Values and error bars reflect mean ± SD.

To address potential safety concerns about AAV-delivered CRISPR genome editing in vivo, the chronic expression of SaCas9 and the risk of AAV vector integration were evaluated. Reverse transcription (RT)–PCR analysis revealed that SaCas9-KKH RNA expression peaked at 4 weeks, decreased at 8 weeks, and became undetectable after 12 weeks in the injected ear (Fig. 7A). Only traces of SaCas9-KKH mRNA were detected in the contralateral uninjected ear. The mRNA level in the contralateral uninjected ear peaked at 4 weeks after injection, which was only 3.33 ± 1.02% compared with that in the injected ear (Fig. 7A). The trace presence of SaCas9-KKH did not induce any InDel in the hair cells (fig. S6D). We also compared the auditory function in both short-term (10 weeks postinjection) and long-term (20 weeks postinjection) periods and found no differences (*P* > 0.5) in ABR (fig. S9, A and C) and DPOAE (fig. S9, B and D) thresholds between the contralateral uninjected ears and the ears from uninjected mice. By Western blotting, we confirmed the presence of SaCas9-KKH protein at 4 weeks postinjection and its absence after 12 weeks (Fig. 7B). We observed more SaCas9-KKH protein at 4 weeks after AAV injection in 3-week-old mice compared with 6-week-old mice (Fig. 7B), suggesting better transduction efficiency in younger mice. These findings collectively show that SaCas9-KKH expression was effectively shut off within 3 months after injection, likely because of the silencing of the CMV promoter, which is known to undergo silencing over time in vivo (47–49).

Another concern is the risk of AAV vector integration into CRISPR-induced DNA breaks (50, 51). To assess AAV vector integration, specific primers were designed to detect the integration of *Mir96* and AAV ITR (Fig. 7C). We first studied the relation between the AAV dosage and AAV vector integration rate. In cultured HEK-293T-*Mir96*^*14C>A*^ cells treated with different AAV2-SaCas9-KKH-sgRNA-4 dosages, AAV vector integration at the double-strand break (DSB) locus became detectable at a dosage of 10^3^ vg per cell (Fig. 7D and fig. S10, A and B). The InDel frequency and the integration ratio were correlated with the increasing AAV dosage. By varying AAV dosages, we identified an optimal dosage range from 10^2^ to 10^3^ vg per cell, where the InDel frequency was near its peak while maintaining low AAV vector integration in HEK cells (Fig. 7D and fig. S10, A and B). These results suggest that fine-tuning the AAV dosage can reduce the potential risk of AAV vector integration while maintaining efficient genome editing.

We assessed AAV vector integration in vivo using designed specific primers (Fig. 7C). We were able to amplify the *Mir96* locus (P1 + P2) DNA and SaCas9-KKH DNA (P3 + P4), but not any *Mir96*-ITR integration DNA (P1 + ITR-R) in the cochlear samples from injected animals (Fig. 7E). Only minimal integration was detected in isolated hair cells from injected animals (Fig. 7F). NGS from isolated hair cells revealed that out of a total of 82,453 reads, the *Mir96* WT allele had 42,991 reads, whereas the remaining reads were associated with *Mir96*^*14C>A*^ reads. Among the *Mir96*^*14C>A*^ reads, InDel-containing reads accounted for 25.7% (Fig. 7G). In addition, there were 102 integration reads, making up 0.26% of the total reads (Fig. 7G). Measurement of miR96 using qPCR showed a decrease in miR96 in the injected ear (89.2 ± 3.0%) compared with the uninjected ear (Fig. 7H), likely because of RNA degradation after genome editing. No change (*P* = 0.87) was observed in the miR96-ITR mRNA (Fig. 7I), indicating the absence of miR96-ITR chimera transcripts after genome editing. These results demonstrate that AAV2-mediated genome editing in the mature cochlea exhibits minimal or negligible vector integration. To explore the possibility of eliminating AAV vector integration, a reduced dose of AAV2-SaCas9-KKH-sgRNA4 (1 × 10^9^ vg per cochlea) was administered. Ten weeks after the injection, no integration of ITR was detected in isolated hair cells from injected ears (fig. S11A), shown by an average InDel frequency of 12.05% (*n* = 3) and ITR integration reads ratio below the background error (fig. S11, B and C). These findings demonstrate that the AAV vector integration rate is correlated with the AAV dosage administrated. We used the dosage to achieve the therapeutic threshold for genome editing therapy while minimizing the AAV vector integration.

CIRCLEseq (circularization for in vitro reporting of cleavage effects by sequencing) is a high-throughput method used to identify off-target sites of CRISPR-Cas9 genome editing tools and was performed to identify potential off-target sites for SaCas9-KKH/sgRNA-4 (52). Only two off-target sites were identified from the mouse genomic DNA (Fig. 7J). Computational predictions were also used to identify potential off-target loci (53, 54). We analyzed the top 10 off-target hits in the mouse genome according to the cutting frequency determination (CFD) score (53) after genome editing (fig. S12, A and B), which includes the two off-target sites identified by the CIRCLEseq analysis. NGS analysis identified one off-target site with a low percentage of InDels (1.97%), whereas the on-target editing efficiency was 84.6% (Fig. 7K). The off-target editing was in the intergenic region (Gm19782-Fam135b), which means that it was unlikely to disrupt any gene function. Using the isolated hair cells from injected mice, we did not detect any off-target InDels (Fig. 7L). Together, these results show that the delivery of AAV2-SaCas9-KKH-sgRNA-4 into *Mir96*^*14C>A/+*^ cells results in minimal off-target modification, and the hearing-related phenotypes are the result of on-target editing.

### A dual-AAV2 system enables targeting multiple human *MIR96* mutations

**Fig. 8:**
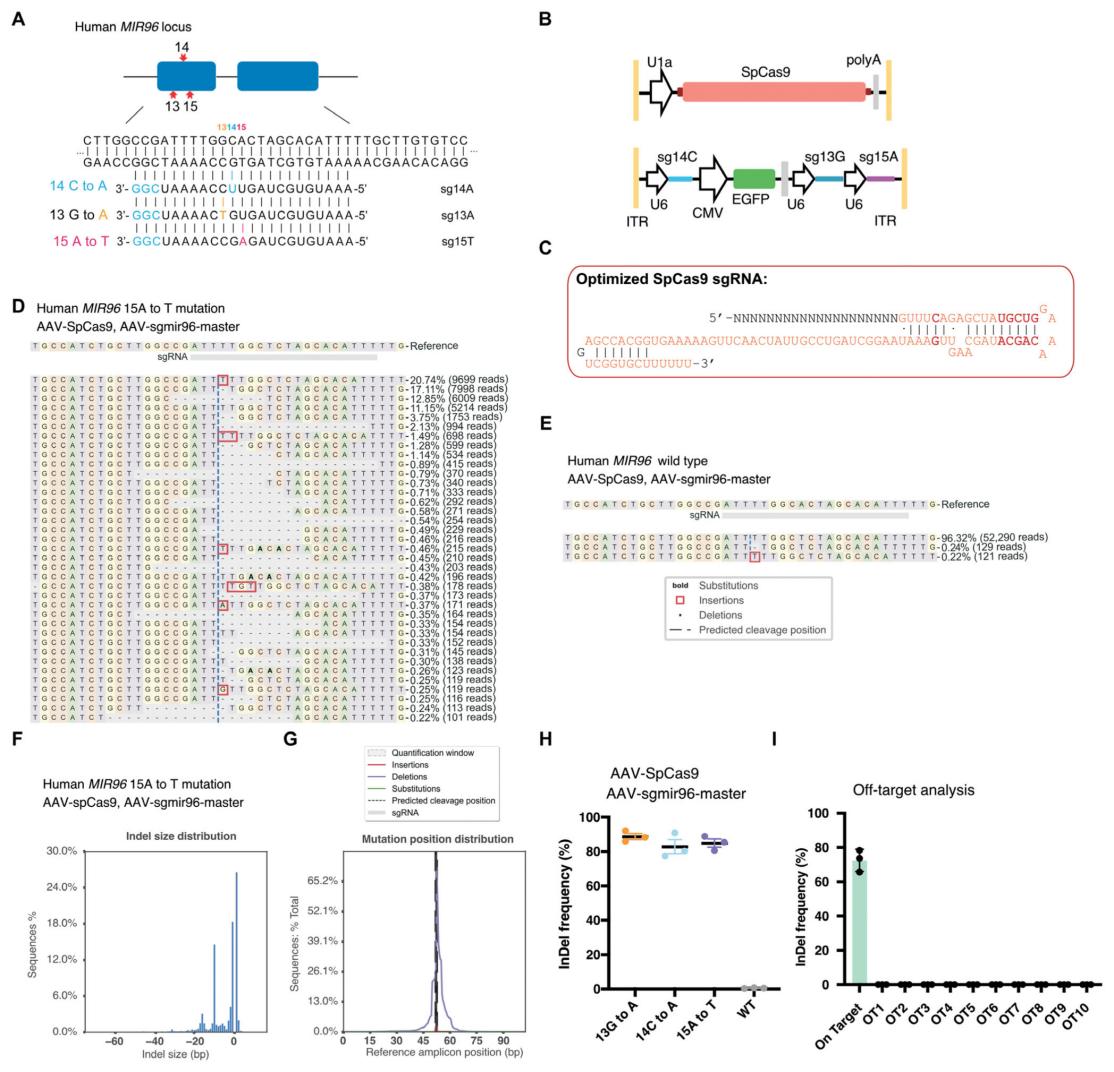
A dual-AAV system carrying multiple gRNAs targeting all known *Mir96* seed region mutations in human cells specifically and efficiently. (**A**) Sequence information of the human *MIR96* locus and the sgRNA design to target three known seed mutations. Red: mutation site. Blue: the PAM nucleotides. (**B**) Schematic view of dual-AAV constructions. One contains the “U1a-SpCas9-polyA” cassette, and the other contains three U6-sgRNA cassettes. (**C**) Sequence of the optimized SpCas9 sgRNA; the bold letters indicate the changes compared with the unmodified sequence. (**D** and **E**) Representative NGS results from SpCas9/sgmiR96-master edited HEK-miR96 (15A to T) cells and SpCas9/sgmiR96-master edited HEK293T WT cells. (**F** and **G**) InDel profiles from SpCas9/sgmiR96-master edited HEK-miR96 (15A to T) cells. Negative numbers represent deletions, and positive numbers represent insertions. Experiments were repeated three times. (**H**) The InDel frequency in the HEK-miR96 mutation cell lines and WT HEK293T cells after genome editing using SpCas9/sgmiR96-master. Each dot represents an independent experiment. Values and error bars reflect mean ± SD. (**I**) Off-target analysis in SpCas9/sgmiR96-master edited HEK-miR96 (15A to T) cells.

Because the *Mir96* seed sequence is 100% conserved across mammalian species, the gRNA designs that target mouse mutations can be directly used to target orthologous human mutations. In addition to the +14C>A mutation, there are two known dominant mutations in the *MIR96* seed region, *MIR96* 13G>A in humans and *MIR96* 15A>T in mice (11, 17). We designed gRNAs for each of the two mutations (Fig. 8A). Cas9 and multiple sgRNA cassettes are too big to fit in a single AAV; thus, we created a dual-AAV set consisting of AAV-U1A-SpCas9-polyA with three NLSs and AAV-sgmiR96-master with sgRNA cassettes to target all three mutations (14C>A, 13G>A, and 15A>T) (Fig. 8, B and C). To validate the dual-AAV set, human cell lines containing the three known *MIR96* seed region mutations were generated using the PiggyBac system, and each cell line was transfected with AAV-U1A-SpCas9-polyA/AAV-sgmiR96-master. NGS revealed robust InDel formation in all three *MIR96* mutation lines, with a similar editing efficiency (Fig. 8, D to G, and fig. S13, A to F). Less than 1% of the reads had InDels detected at the *MIR96* locus (Fig. 8E). The data support specific disruption of each mutant allele by its corresponding gRNA, with an editing efficiency above 80% (Fig. 8H). We also analyzed the top 10 potential off-target sites in the human genome, and NGS showed no InDel after Cas9/sgRNA transfection (Fig. 8I and fig. S13G). These findings suggest that the combination of AAV-U1A-SpCas9-polyA/AAV-sgmiR96-master can effectively target each of the three known human *MIR96* mutations and holds promise for treating dominant hearing loss caused by *MIR96*-related mutations. The design of the AAV-sgmiR96-master system expands the targeting scope and simplifies the efficacy and safety evaluation of the AAV delivery system.

## Discussion

With the goal of developing a clinical treatment for human genetic hearing loss of DFNA50, we conducted genome editing in adult *Mir96*^*14C>A/+*^ cochleae and successfully improved auditory function long term. miRNAs are involved in multiple physiological and pathological inner ear processes (55) and have been linked to different types of hearing loss, including deafness related to hair cell development (8, 56), age-related hearing loss (57), noise-induced hearing loss (58), and inner ear inflammation (59). In humans, heterozygous *MIR96* mutations result in delayed-onset nonsyndromic progressive hearing loss, indicating the potential loss of sensory hair cell identity and subsequent dysfunction in the mature cochlea, thereby offering an opportunity for genetic intervention in patients with DFNA50.

We designed and screened for genome editors by testing editing efficiency in primary fibroblasts and identified SpCas9-sgRNA-1 and SaCas9-KKH-sgRNA-4, respectively. We further optimized sgRNAs to improve the editing efficiency. AAV2 was chosen as the delivery vehicle for its efficient transduction in adult IHCs and OHCs (39, 40). We conducted injections of AAV2 into adult mice across various age groups. Genetic disruption of the *Mir96*^*14C>A*^ allele by NHEJ leads to improved survival of hair cells and the preservation of auditory function in *Mir96*^*14C>A/+*^ mice. The therapy was effective in improving long-term hearing in both presymptomatic and symptomatic stages of hearing loss in *Mir96*^*14C>A/+*^ mice. We furthermore observed only transient expression of SaCas9-KKH in the inner ear, presumably because the CMV promoter became silenced over time in vivo. This temporary expression provides a controlled and regulated editing process, which enhances the safety and precision of the therapy. In addition, we demonstrated that fine-tuning the dosage of AAV helps minimize the risk of AAV-mediated integration and improves the safety profile of the therapy. As a result, we did not detect any off-target effects. Combined, these findings greatly enhance the safety profile of in vivo inner ear genome editing therapy. The study also introduces a dual-AAV system capable of targeting all known human *MIR96* seed region mutations, making it a promising approach to treat DFNA50 due to different *MIR96* dominant mutations. Our study highlights the editing efficiency, safety, and long-term therapeutic efficacy of genome editing in treating hearing loss due to *Mir96* mutations in adult animal models and provides a promising path for clinical applications.

The selection of the most suitable editor and delivery vehicle is crucial to developing a genome editing therapy for genetic disorders. Here, we chose Cas9 nucleases to disrupt the mutated allele of *Mir96*^*14C>A/+*^ because of its gain-of-function inheritance pattern (18) so that the resulting InDels would abolish the expression of the disease-causing mutant *Mir96*. Although prime editing has the potential to correct all the *Mir96* mutations, our in vitro data showed a modest efficiency at the *Mir96* locus, suggesting that further optimization is needed for improved editing efficiency. AAV2 was selected as the preferred delivery vehicle for CRISPR because of its specific targeting of IHCs and OHCs in the cochleae of adult mice (39). In addition, the AAV2 serotype has been used in the US Food and Drug Administration–approved human gene therapies (60, 61).

One of the major concerns associated with AAV-mediated CRISPR genome editing in vivo is the potential for off-target editing caused by prolonged expression of Cas9/sgRNA (62). High-fidelity versions of CRISPR systems (35, 63, 64) can be applied to improve specificity. For example, we have demonstrated that LZ3 Cas9 can target the *Mir96*^*14C>A*^ allele robustly and specifically. In addition, minimizing genome editing in unintended tissues and cell types is also an important factor in the evaluation of the safety of CRISPR-based therapeutics. AAV2 can specifically deliver Cas9/sgRNA into hair cells through local injection, reducing the likelihood of editing unwanted cells or tissues. Besides, the use of the CMV promoter for SaCas9-KKH expression helps minimize off-target editing because it tends to become silenced over time in vivo (47–49, 51, 65–68). There are studies showing that transgenes tend to become silencing over time in nonhuman primates and human embryonic stem cells and derivates (69–71). We demonstrated here that CMV-driven SaCas9-KKH was only transiently expressed and silenced in 3 months in cochleae. Another concern is the risk of vector integration at DNA DSBs (50, 51). The integration ratio of AAV varies across different genomic loci, tissues, and cell types (50). By focusing on hair cell–limited genome editing, we were able to reduce the occurrence of vector integration to a negligible level. Furthermore, we showed that the probability of vector integration is correlated with the AAV dosage, underscoring the importance of fine-tuning the amount of AAV delivered to maximize efficacy and minimize potential integration, which will likely be important for clinical development.

An age-dependent rescue of the auditory function is demonstrated in our study. Comparing three age groups of injection at 3, 6, and 16 weeks of age, the earliest injection at 3 weeks achieved the best recovery by the reduction in the ABR and DPOAE thresholds at a comparable postinjection age. Western blotting also revealed higher SaCas9-KKH expression in younger mice, indicating better transduction efficiency. This suggests that genome editing therapy at an earlier stage may lead to increased SaCas9-KKH expression, which is likely to contribute to improved rescue in the auditory function. Even for the injection at 3 weeks, the effect of AAV-mediated editing and subsequent restoration of target gene expression will likely lag behind when hearing loss has started, yet hearing rescue was more robust. The data illustrate that for editing therapy of *Mir96*^*14C>A/+*^ mice, the window of opportunity for intervention is before the hearing loss has become too severe to be rescued, as shown by the lack of efficacy by injection in 16-week-old *Mir96*^*14C>A/+*^ mice. This is expected as hair cells start to degenerate or are lost over time in *Mir96*^*14C>A/+*^ mice, and later intervention will not be able to repair severely damaged hair cells or replace the lost hair cells. In patients with DFNA50, hearing loss starts postlingual and becomes severe over decades (11), suggesting a relatively long period within which editing therapy could be efficacious. This information, combined with our robust hearing rescue in the *Mir96*^*14C>A/+*^ mice, strongly supports the development of the work toward the clinic.

There are limitations in our study. We have obtained an in vivo editing efficiency of 16 to 25%. It is likely that further improved editing efficiency will result in better treatment efficacy. We used AAV-mediated delivery with Cas9 expression that is transitory without major genome integration by a therapeutically beneficial dose in mice. For the applications in humans, the system has to be further evaluated, including the testing in nonhuman primates and human inner ear specimen. Transient nonviral delivery systems such as nanoparticles will greatly improve the safety of editing therapy.

We established a feasibility of in vivo genome editing as a viable approach for treating hearing loss caused by genetic mutations in adult animals, offering insights into the potential clinical applications of this technology for treating inherited hearing disorders. Recently, we and others have successfully conducted an otoferlin gene therapy clinical trial to restore hearing in children with recessive deafness DFNB9 based on the treatment study in the mouse models (72, 73), which demonstrated the translatability from mouse models to humans. The robust hearing rescue and strong safety profile of our editing study in *Mir96*^*14C>A/+*^ mice should allow for further testing of this strategy in future preclinical and clinical trials.

## Materials and Methods

### Study design

Our study focused on hearing loss caused by mutations in *microRNA-96* and the potential use of genome editing for treatment. The study used CRISPR genome editing techniques, specifically AAV-mediated delivery of the editing complex, to target and disrupt a dominant *MIR96* mutation (*Mir96 14C>A*) in adult mice (*Mir96*^*14C>A/+*^) with hearing loss. We evaluated the hearing phenotype using ABR and DPOAE functional test in two procedures: evaluation of short- and long-term efficacy and establishment of a time window for the treatment to result in sustained hearing preservation. We also addressed safety concerns, including the duration of Cas9 expression and the risk of AAV vector integration, and demonstrated the transient expression of Cas9, reducing potential off-target effects and integration risks. The morphology of OHCs and IHCs was evaluated with antibody staining and SEM. Details on the use of cell line and animal in different experiments can be found in the corresponding sections of Materials and Methods. Sample sizes were chosen on the basis of previous studies in the field without statistical prediction. Animals were randomly assigned to experimental groups, and researchers were blinded during ABR and DPOAE functional test, immunostaining, and SEM, but not others. All procedures for generating the mutant mouse were carried out in accordance with UK Home Office regulations and the UK Animals (Scientific Procedures) Act of 1986 (ASPA) under UK Home Office licenses, and the study was approved by the Wellcome Sanger Institute Ethical Review Committee. All studies involving animals were approved by the HMS Standing Committee on Animals and the Mass Eye and Ear Infirmary Animal Care and Use Committee (IACUC). For all experiments, the number of replicates, statistical tests used, and *P* values are reported in the figure and legends.

### Animals and surgery

The *Mir96*^*14C>A*^ allele (official symbol *Mir96*^*tm3.1Wtsi*^, referred to here as *Mir96*^*14C>A*^) was generated at the Wellcome Sanger Institute by targeting of the BEPD0003_D07 ES cell of C57BL/6N origin and was maintained on the same genetic background. After shipping, all animals were bred and housed in Mass Eye and Ear Infirmary. All mice were housed in a room maintained on a 12-hour light/dark cycle with ad libitum access to standard rodent diet. The *Mir96*^*14C>A*^ mice are available from the European Mutant Mouse Archive (strain number BEPD0003_D07).

*Mir96*^*14C>A/+*^ and WT mice of either sex were anesthetized using intraperitoneal injection of ketamine (100 mg/kg) and xylazine (10 mg/kg). The postauricular incision was exposed by shaving and disinfected using 10% povidone iodine. The AAV2-CMV-SaCas9-KKH-sgRNA4 of two titers was injected into the inner ears of *Mir96*^*14C>A/+*^ mice. The AAV2-GFP was injected into the inner ears of WT mice. The total volume for each injection was 1 to 1.2 μl of virus per cochlea.

### Plasmid construction

pMax-SpCas9 was constructed on the basis of previously published sequences (74, 75); the U6-sgRNA sequence was also obtained from Addgene 48138 (76), and other Cas9 nuclease cDNAs, including scCas9++ (Addgene 155011) (32), SaCas9-KKH (Addgene 70707) (33), sauriCas9 (Addgene 135964) (34), and LZ3 Cas9 (Addgene 140561) (35), were acquired from Addgene. Vectors for in vitro screening were constructed through Gibson assembly (NEB, E2611S). SaCas9-KKH AAV plasmid (fig. S14) was constructed on the basis of a previously published sequence (Addgene 61591) (77) by replacing SaCas9 with SaCas9-KKH. AAV-U1a-opti-SpCas9-PA (fig. S15) was based on Addgene 121507 (78). AAV-sgmir96-Master (fig. S16) is based on pAAV-U6-sgRNA-CMV-GFP (Addgene 85451) (79). Plasmids encoding recombinant AAV (rAAV) genomes were cloned by Gibson assembly. All plasmids were purified using Plasmid Plus Miniprep or Maxiprep kits (Qiagen).

### Hair cell isolation and NGS analysis

Cochleae were harvested with the sensory epithelia dissociated using needles under the microscope (Axiovert 200M, Carl Zeiss). Inner ear tissue was immersed in 1 μM FM 1-43FX (Thermo Fisher Scientific, F35355) dissolved in Dulbecco’s phosphate-buffered saline (DPBS) (Thermo Fisher Scientific) for 15 s at room temperature in the dark, then washed by DPBS. The sensory epithelia were treated with 100 μl of 0.05% trypsin-EDTA (Thermo Fisher Scientific, 25300054) for 10 to 20 min. During incubation, the tissue was carefully dispersed into small cell clusters or single cells using a 200-μl Eppendorf pipette tip. Cells were then transferred into a six-well plate and placed under a fluorescent microscope (ZEISS) equipped with a camera. Cells with FM 1-43FX dye were collected by a 10-μl Eppendorf pipette tip. Above 300 cells were collected from a single cochlea. Hair cells were transferred into a 200-μl PCR tube and centrifuged for 5 min, 200 rcf. Supernatants were carefully discarded, and the isolated hair cells were lysed by 5 μl of QuickExtract DNA Extraction Solution (Lucigen) and incubated at 65°C for 6 min and then at 98°C for 3 min. All 5 μl of the cell lysis was used for Genomic PCR amplification using NEBNext Ultra II Q5 Master Mix (NEB, M0544S). The PCR program is as follows: 1 cycle: 98°C, 5 min; 42 cycles: 98°C, 15 s; 60°C, 20 s; and 72°C, 10 s; 1 cycle: 72°C, 4 min; 4°C, hold. PCR product visualization, purification, and NGS analysis are the same as that described above (80, 81).

### Hearing function testing

*Mir96*^*14C>A/+*^ mice of either sex were anesthetized using intraperitoneal injection of ketamine (100 mg/kg) and xylazine (10 mg/kg). For ABR measurements, subcutaneous needle electrodes were inserted at the vertex, ventral edge of the pinna (active electrode), and a ground reference near the tail. The mice were placed in a sound-proof chamber and exposed to 5-ms tone pips delivered at a rate of 35 per second. The response was amplified 10,000-fold, filtered with a band pass of 100 Hz to 3 kHz, digitized, and averaged using 1024 responses at each SPL. The sound level was elevated in 5-dB steps from 20 dB up to 90 dB SPL, with stimuli ranging from 5.66 to 45.24 kHz frequencies (in half-octave steps). The “threshold” and wave 1 amplitude were identified as described previously (19, 20). During the same recording session, DPOAEs were measured under the same conditions as for ABRs. Briefly, two primary tones (f2/f1 = 1.2) were set with f2 varied between 5.66 and 45.24 kHz in half-octave steps. Primaries were swept from 20 dB SPL to 80 dB SPL (for f2) in 5-dB steps. Thresholds required to produce a DPOAE at 5 dB SPL were computed by interpolation as f2 level.

### AAV vector integration assay

HEK-293T-*Mir96*^*14C>A*^ cells were treated with AAV2-CMV-SaCas9-KKH-sgRNA4 of different dosages from 1 to 10^7^ genomic copies per cell. Control cells were transduced with AAV2-CMV-SaCas9-KKH-sgCtrl, 10^5^ genomic copies per cell. Cells were collected 7 days later, and genomic DNA was isolated. Primers P1-F/ITR-R were used for detecting of AAV vector integration. For in vivo integration detection, primers P1-F/P2-R and primers P1-F/ITR-R were used to amplify genomic fragment from isolated hair cells, and then PCR products were merged together for NGS analysis. Primers premir96-F/ITR-R were used in the qRT-PCR study to detect mir96-ITR transcription. All primers used for AAV vector integration assay are listed in table S1.

### Statistical analysis

The number of biological and technical replicates and parameters are indicated in the corresponding figure legends. Statistical analyses were performed using GraphPad Prism 8. Student’s *t* test was used to calculate statistical significance between two groups, and two-way analysis of variance (ANOVA) tests were used for differences between three groups. The *P* values <0.05 were considered as significantly different. ****P < 0.0001, ***P < 0.001, **P < 0.01, and *P < 0.05.

## Supplementary Material

Supplementary material

## Data Availability

All data associated with this study are present in the paper or the Supplementary Materials. AAV genome sequences are provided in the Supplemental Data (figs. S14 to S16). The plasmids used in this study can be made available from the corresponding author under a material transfer agreement with Massachusetts Eye and Ear Infirmary, Harvard Medical School. All NGS data are available with the Sequence Read Archive (SRA) accession number PRJNA1088125.
