# Supplementary material for: Targeted genome editing restores auditory function in adult mice with progressive hearing loss caused by a human microRNA mutation
Source: Sci Transl Med. Author manuscript; Available in PMC 2024 Jul 31. (PMC7616320; doi:10.1126/scitranslmed.adn0689)
Supplement: Supplementary material [file EMS197763-supplement-Supplementary_material.pdf]

Science Translational Medicine
AAAS

Supplementary Materials for

Targeted genome editing restores auditory function in adult mice with progressive hearing loss caused by a human microRNA mutation

Wenliang Zhu *et al.*

Corresponding author: Zheng-Yi Chen, zheng-yi_chen@meei.harvard.edu

Sci. Transl. Med. **16**, eadn0689 (2024)
DOI: 10.1126/scitranslmed.adn0689

The PDF file includes:

Supplementary Materials and Methods
Figs. S1 to S17
Tables S1 to S3
References

Other Supplementary Material for this manuscript includes the following:

Data file S1
MDAR Reproducibility Checklist

MATERIALS AND METHODS

AAV production

AAV vectors were produced by Mass eye and ear infirmary vector core (Boston, MA, USA). AAV plasmids containing “CMV-SaCas9-KKH-PA” cassette, “U6-sgRNA-4” or “U6-sgCtrl” cassette were sequenced before packaging (MGH DNA Core, complete plasmid sequencing) into AAV2/2. Vector titer was 5.76×10^{12} vg/ml for SaCas9-KKH/sgRNA-4 and 4.55×10^{12} vg/ml for SaCas9-KKH/sgCtrl as determined by qPCR specific for the inverted terminal repeat of the virus.

Isolation and culture of Primary Fibroblasts from Mice

Mir96$^{14C>A/+}$, Ai14 and wild type mice were euthanized and cleaned with 70% ethanol. The dorsal skin (about 1 cm diameter) of the mice was collected and rinsed with DPBS, subcutaneous fat was removed by forceps. Subsequently, the samples were cut into small fragments and incubated with Dispase II (Sigma-Aldrich, USA) for overnight at 4 °C. The dermal layers of the skin were separated using forceps and further subjected to incubation with type I collagenase (1 mg/ml Gibco, USA) for 2 hours at 37 degrees. The resulting cell suspension was strained using a 40-micron strainer and centrifuged at 950 rpm to obtain the cell pellet. The cell pellet was seeded in T-75 flask containing DMEM high glucose media (Gibco, USA) containing 10 % FBS (Gibco, USA). Fibroblasts were cultured for about 2–3 days to reach about 90% confluence, then passaged in T75 flasks with TrypLE Express and cultured in DMEM: F12 medium (ThermoFisher) with 10% fetal bovine serum (FBS) supplemented with GlutaMax (ThermoFisher).

Construction of *Mir96*$^{14C>A}$ cell line using PiggyBac

Mouse *Mir96*$^{14C>A}$ (0.6 kb) harboring the +14C>A mutation was amplified by PCR from *Mir96*$^{14C>A/14C>A}$ mouse genomic DNA. The PCR products were cloned into the PiggyBac donor backbone (PB-CAG-mNeonGreen-P2A-BSD-polyA) using Gibson Assembly. The constructed donor plasmid was co-transfected with PiggyBac transposon vector (PB210PA, System Biosciences) into HEI-OC1 cells. Cells were cultured and selected in the medium containing 10 µg/mL Blasticidin for 2 weeks. For human *MIR96* +14C>A fragment, mutation was introduced

by PCR and cloned into the same PiggyBac donor backbone using Gibson Assembly. Cells were transfected by PiggyBac plasmids and selected by Blasticidin for 2 weeks. Successful insertion was confirmed by PCR and sequencing analysis. Clones from the positive *Mir96*$^{14C>A}$ selection were expanded for subsequent studies.

Genome editing in vitro

We performed nucleofection using LONZA 4D-Nucleofector (*80*). Cells were digested by Trypsin-EDTA (0.05%) (Thermo Fisher) and future dispersed into single cells. 100,000 cells were resuspended in 20μl P3 reagent of the P3 Primary Cell 4D-Nucleofector® X Kit S (Lonza V4XP-3032). 1 μg total plasmid was used for a single nucleofection event and nucleofected by program EH-100. Cells were not sorted. 5 days after the nucleofection, cells were lysed by QuickExtract™ DNA Extraction Solution (Lucigen) to extract genomic DNA.

Next generation sequencing (NGS) and InDel analysis_

Treated cells (in vitro genome editing) or tissues (in vivo genome editing) were collected after genome editing. Genomic PCR was carried out using NEBNext® Ultra™ II Q5® Master Mix (NEB, M0544S) to amplify the *Mir96* locus, 400-800 ng of purified PCR product were used for next generation sequencing (NGS), samples were sequenced by the Massachusetts General Hospital Center for Computational and Integrative Biology DNA Core and analyzed to detect CRISPR variants from NGS reads using CRISPResso2 (http://crispresso.pinellolab.org/submission) as instructed (*81*). For cochlea samples, each NGS data represents a unique sequencing reaction from a combination of multiple cochleae, as indicated in the figure legends. The sgRNA protospacer sequences can be found in table S3.

Immunofluorescence staining

Cochleae, both injected and non-injected, were harvested. Temporal bones were fixed in 4% paraformaldehyde at 4°C overnight and subsequently decalcified in 120 mM EDTA for a week. Then the organ of Corti was dissected for whole-mount immunofluorescence. The dissected tissues were blocked with a blocking solution (PBS with 8% donkey serum and 0.3-1% Triton X-100) for 1 hour at room temperature. Subsequently, the specimens were subjected to overnight incubation with the primary antibodies: anti-MYO7A (#25-6790, Proteus BioSciences), anti-GFP (ab13970, Abcam). After three rinses with PBS, the tissues were incubated with the

corresponding secondary antibodies for 1 hour. Finally, all specimens were mounted with VECTASHIELD antifade mounting medium containing DAPI (VECTOR LABORATORIES, #H-1200). Images were taken with a Leica SP8 confocal laser scanning microscope (Leica Microsystems, Germany). For hair cell counting, MYO7A-positive hair cells per 100μm length were calculated in the apex, middle, and middle-base turns of cochleae. We counted from three independent cochleae.

Scanning Electron Microscopy

Following cochlea dissection, the harvested tissues were placed in 2.5% glutaraldehyde solution in 0.1 M cacodylate buffer (EMS) supplemented with 2 mM CaCl2. The immersion was performed for 1.5-2 hours at room temperature on a tissue rotator. Subsequently, the samples were rinsed three times with distilled water. The samples underwent three 10-minute rinses with 0.1 M sodium cacodylate buffer. Next, they were treated with 1% osmium tetroxide for a duration of one hour, followed by another three 10-minute rinses with distilled water. The samples were then subjected to treatment with saturated thiocarbohydrazide in distilled water for 30 minutes. This cycle of treatments (one-two-one-two-one) was repeated.

For dehydration, the samples were transferred to 20 ml scintillation vials containing 2 ml of distilled water. The vials were supplemented with 50 μL of 100% ethanol, with the volume doubled every 10 minutes until the vial was full. At that point, the samples were transferred to 100% ethanol. Subsequently, the samples were dried using liquid CO2 in a Tousimis Autosamdri 815 to reach the critical point. Finally, the samples were mounted onto aluminum specimen stubs using carbon tape, spatter-coated with a 4.5 nm layer of platinum using a Leica EM ACE600 and analyzed using a Hitachi S-4700 scanning electron microscope (SEM).

RNA isolation and qRT-PCR

Total RNA was extracted from inner ear tissue using the ReliaPrep RNA Tissue Miniprep System (Promega, z6111). The first-strand cDNA was produced using ProtoScript® II First Strand cDNA Synthesis Kit (NEB, E6560s) with random primers, following the manufacturer's instructions. STEM-LOOP qRT-PCR were used to measure *Mir96* level, first-strand cDNA was produced using *Mir96* specific primers (rtmir96: CTCAACTGGTGTCGTGGAGTCGGCAATTCAGTTGAGCAAAAATGTG). Real time quantitative PCR was performed using Power SYBR Green PCR Master Mix (Applied

Biosystems, 4368708) on the ABI QuantStudio 3 Flex Real-Time PCR System (Applied Biosystems). qmiR96-F: TCGGCAGGTTTGGAACTAGCAC; qmiR96-R: CTCAACTGGTGTCGTGGA.

Western blotting

After dissection of cochleae, inner ear tissue samples were lysed using RIPA Lysis and Extraction buffer (ThermoFisher, 89900) containing protease inhibitor (ThermoFisher) for 30 min on ice. Protein was quantified and 80 μg of each lysate were loaded per lane of a NuPAGE™ 4-12% Bis-Tris Protein Gel (Thermo Fisher Scientific, NP0335PK2). Samples were separated on 200V for 35 min in the mini gel tank (ThermoFisher). Protein were then transferred to Nitrocellulose Blotting Membranes (PALL, P66485). The membranes were blocked in 5% evaporated milk and followed by incubating with primary antibodies overnight at 4 ºC (Anti-HA: Cell Signaling Technology, 3724S; anti-GAPDH: Thermo, MA5-15738). After three rinses with TBST, NC membranes were incubated with HRP conjugated secondary antibodies (Thermo Fisher Scientific). Protein bands were visualized using SuperSignal West Pico PLUS Chemiluminescent Substrate (ThermoFisher, 34577) and blot image were captured by ChemiDoc Imaging system (BioRad). Uncropped images can be found in fig. S17.

Off-target analysis

To identify off-target sites, the CIRCLE-seq was performed essentially as previously described with the minor modifications. Briefly, 50 μg genomic DNA were purified from HEI-OC1 cells. Genomic DNA was sheared (NEB Ultra II FS kit, E7805) and circularized. In vitro cleavage reaction of circularized genomic DNA is performed with SaCas9-KKH/sgRNA-4. Then the sequencing libraries were prepared and sequenced on an Illumina Miseq as previously described (*82*). Off-target sites were identified with the standard pipeline.

Top10 potential off-target sites were listed according to the CFD score (*83*). We designed PCR primers on the two flanks of each sgRNA target sequences, amplified fragments were purified for NGS to identify whether there is any off-target mutation. All primers used for off-target analysis were listed in table S2.

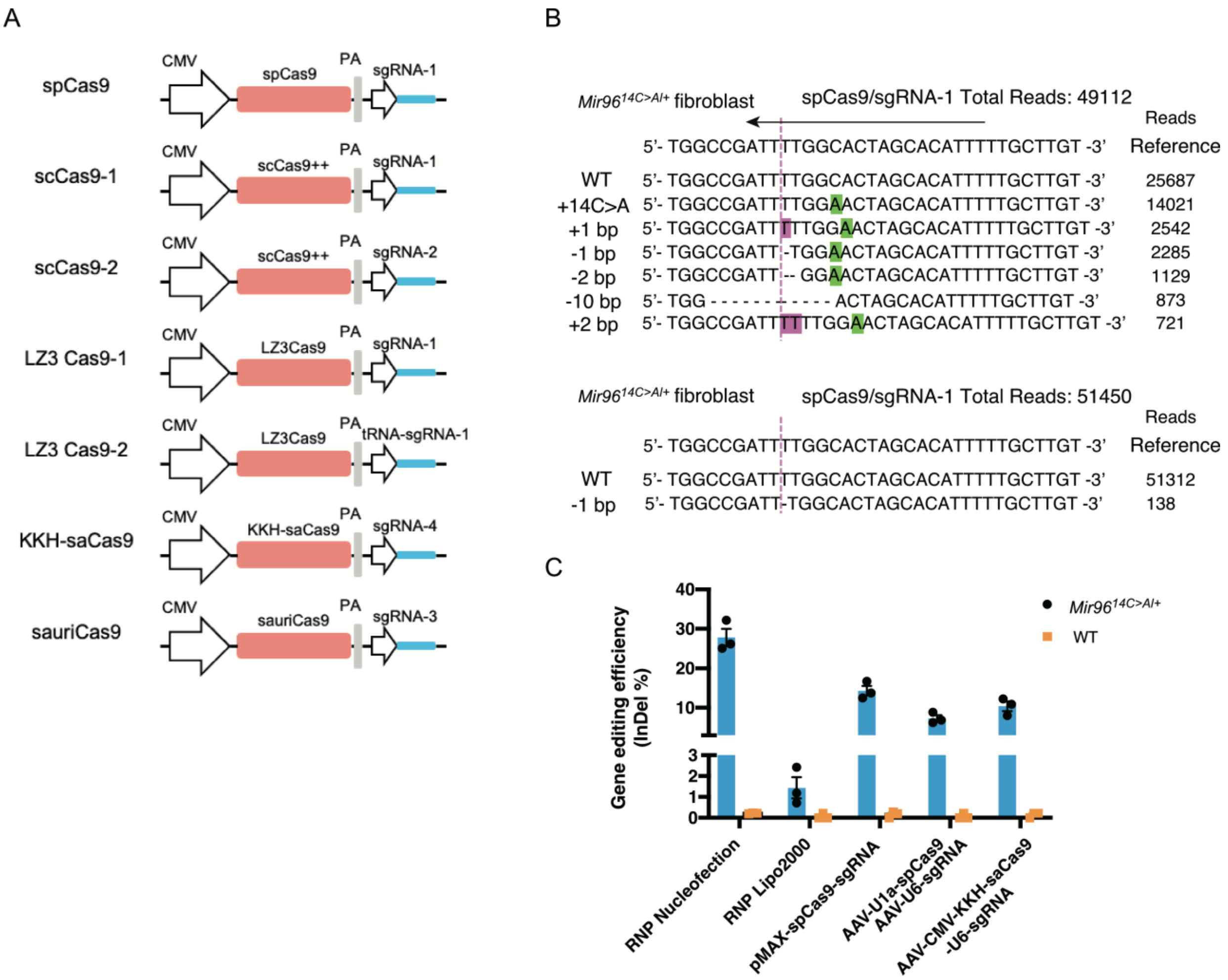

Fig. S1. Targeting *Mir96*$^{14C>A}$ mutation with different CRISPR nuclease systems. (A), Schematic diagram of plasmid constructions for different CRISPR systems used for in vitro screening. **(B)**, Representative reads of the NGS from spCas9/sgRNA-1 edited *Mir96*$^{14C>A/+}$and wild-type primary fibroblasts. Magenta Dotted Lines indicate the double stranded DNA cutting site. Green indicates the mutant nucleotide. **(C)**, The indel frequency in *Mir96*$^{14C>A/+}$ and wild-type primary fibroblasts after editing by different delivery methods and editors. Each dot represents an independent experiment. Values and error bars reflect mean ± SD.

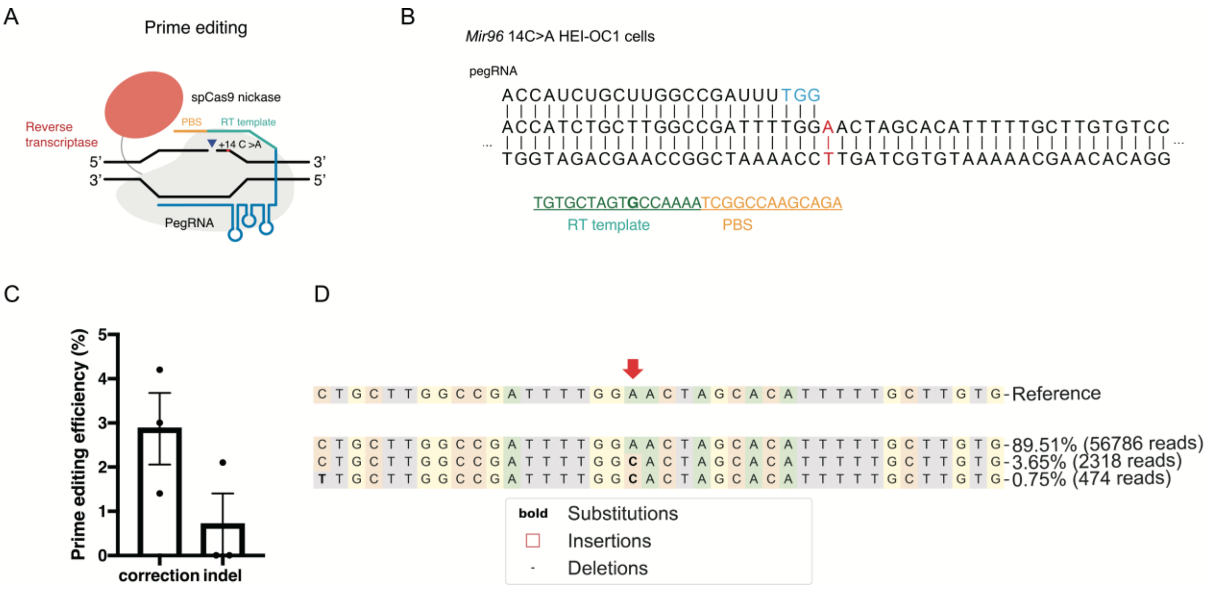

Fig. S2. The correction of *Mir96*$^{14C>A}$ mutation using prime editing in human cells. (A), Schematic overview of the prime editing system. **(B)**, Sequence of the *Mir96* +14 C>A mutation locus and the prime editing design. The mutation nucleotide in the *Mir96*$^{14C>A}$ allele is displayed in red. The protospacer adjacent motifs (PAMs) nucleotides of the pegRNA are displayed in blue. **(C)**, A to C correction frequency after prime editing. Each dot represents an independent experiment. Values and error bars reflect mean ± SD. **(D)**, Representative NGS result after prime editing, showing the corrected reads. Red arrow indicates the *Mir96* +14 C>A mutation nucleotide. Reference sequence is the mutant allele.

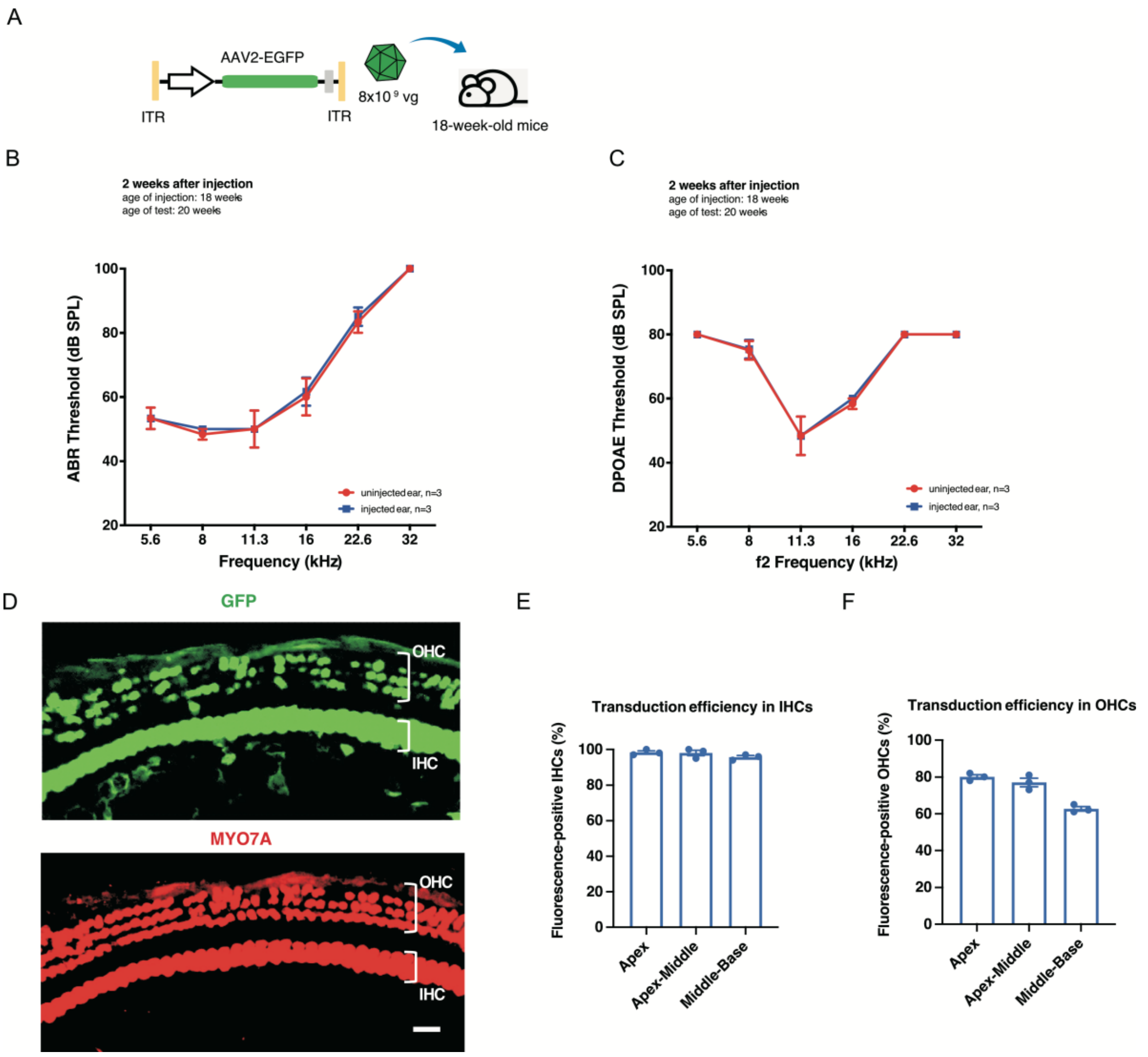

Fig. S3, GFP delivery into the hair cells of mature cochlea using AAV2. (A), Schematic diagram of AAV2 constructions for GFP delivery used for in vivo delivery into adult mouse cochleae. (**B and C**), ABR thresholds (**B**) and DPOAE thresholds (**C**) after AAV2-GFP injected (blue), compared to uninjected ears (red). Values and error bars reflect mean ± SEM. **(D),** Representative confocal images of AAV2 transduction in the apical region of adult cochlea. Hair cells were labeled with MYO7A (red) and AAV was labeled with GFP (green). Scale bar, 20µm. **(E** and **F),** Transduction efficiency of IHCs (**E**) and OHC (**F**) by AAV2-GFP across different turns of the cochlea (Apex, Apex-Middle, and Middle-Base). Time of imaging: 22 weeks of age. Values and error bars reflect mean ± SD, n=3. Each dot represents an independent experiment.

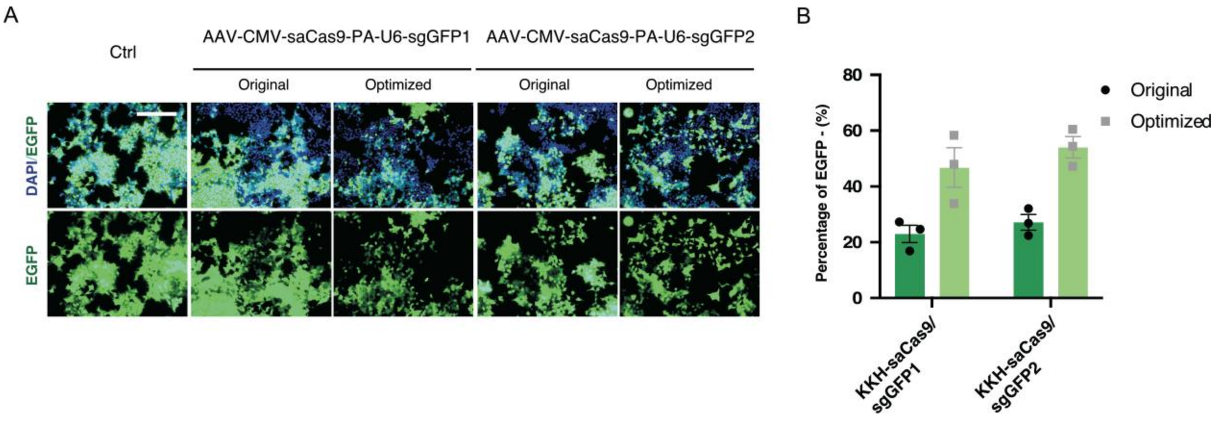

Fig. S4, Genome editing efficiency of optimized AAV vectors. (A), Representative fluorescence images of HEK-GFP cells after gemome editing of unmodified and optimized SaCas9-KKH/sgRNA targeting GFP. GFP negative cells are the edited cells. **(B)**, Editing efficiency shown by the percentage of GFP negative cells after genome editing. Each dot represents an independent experiment. Values and error bars reflect mean ± SD.

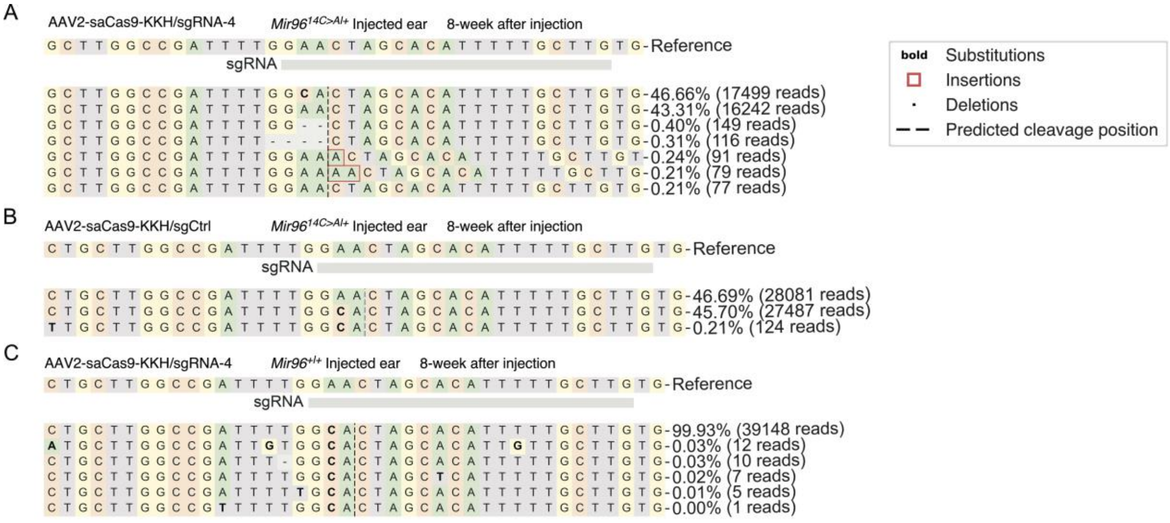

Fig. S5, Genome editing at *Mir96*$^{14C>A}$ locus in adult *Mir96*$^{14C>A/+}$ mice. (A to **C)**, Representative NGS results of AAV2-SaCas9-KKH-sgRNA-4 injected ears from *Mir96*$^{14C>A/+}$ mice and *Mir96*$^{+/+}$ mice, and AAV2-SaCas9-KKH-sgCtrl injected ears from *Mir96*$^{14C>A/+}$ mice. Reference sequence is the mutant allele.

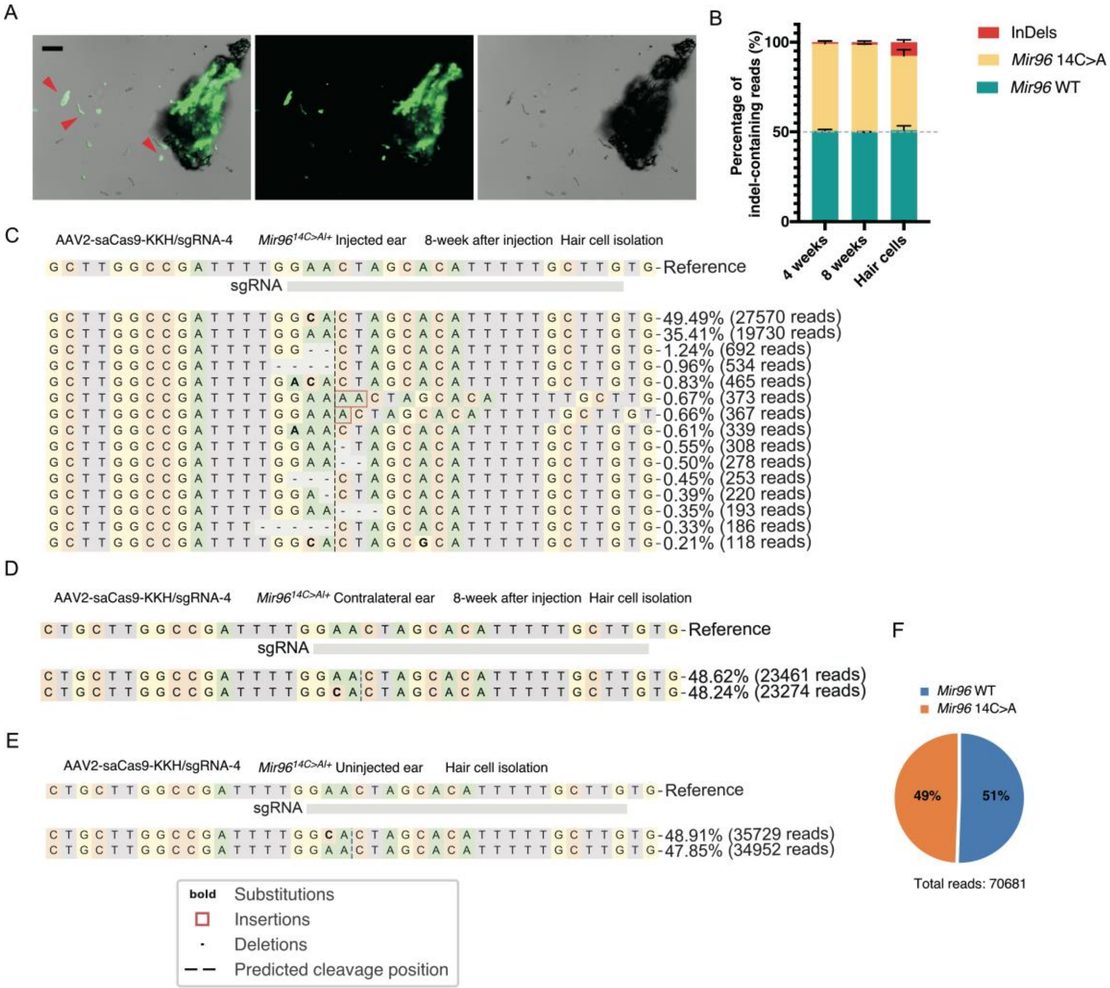

Fig. S6, Precise editing efficiency at *Mir96*$^{14C>A}$ locus in adult *Mir96*$^{14C>A/+}$ mice. (A), Representative images of FM1-43FX labeled hair cells after digestion of cochlear tissues. FM1-43FX labeled hair cells are shown in green. Arrowheads point to the FM1-43FX^{+} hair cells that were picked for NGS. **(B)**, Quantification of the percentage of indel-containing reads in the NGS results of AAV2-SaCas9-KKH-sgRNA-4 edited cochlea samples and isolated hair cells lysis. Values and error bars reflect mean ± SD, n=3. **(C** to **E)**, Representative NGS results of the isolated hair cells from AAV2-SaCas9-KKH-sgRNA-4 injected (**C**), contralateral (**D**) and uninjected ears (**E**) of *Mir96*$^{14C>A/+}$ mice. Reference sequence is the mutant allele. (**F**), Pie chart showing the percentage of *Mir96* wild-type allele reads (blue) and14C>A reads (orange) in the NGS results from uninjected ear.

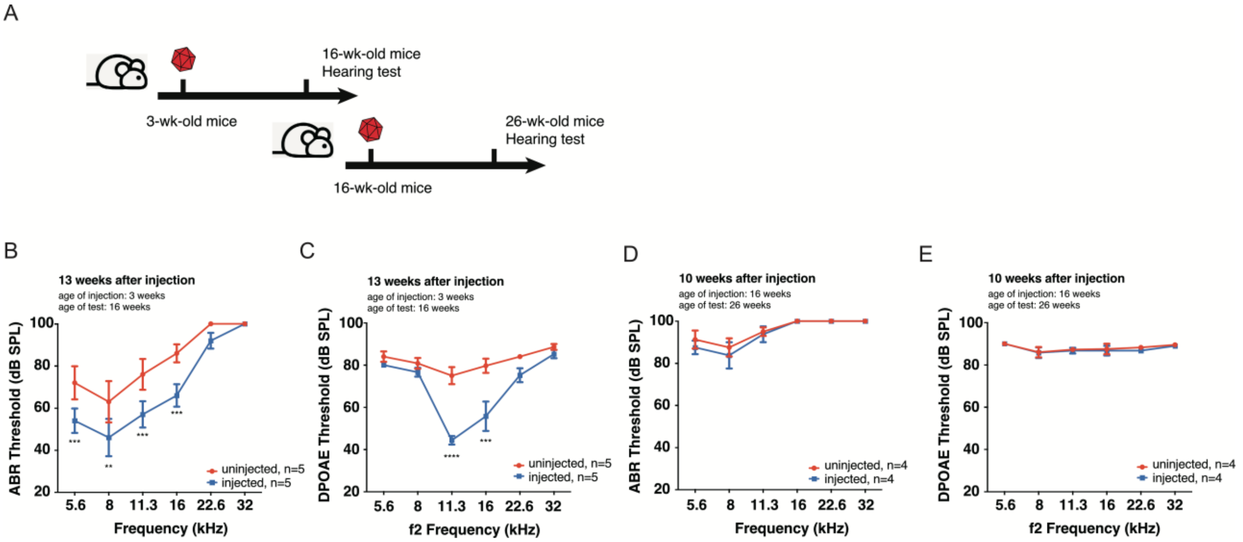

Fig. S7, Age-dependent treatment outcome by editing in *Mir96*$^{14C>A/+}$ mice. (A), The experimental design of inner ear injections of AAV2-CMV-SaCas9-KKH-sgRNA-4 in 3-week-old and 16-week-old *Mir96*$^{14C>A/+}$ mice followed by hearing test. **(B** to **E),** Effects on auditory function restoration. ABR **(B** and **D)** and DPOAE **(C** and **E)** thresholds in *Mir96*$^{14C>A/+}$ mice treated with AAV2-CMV-SaCas9-KKH-sgRNA-4 ears (blue) and untreated ears (red) at 16 weeks age **(B** and **C)** and 26 weeks age **(D** and **E)**. Values and error bars reflect mean ± SEM.

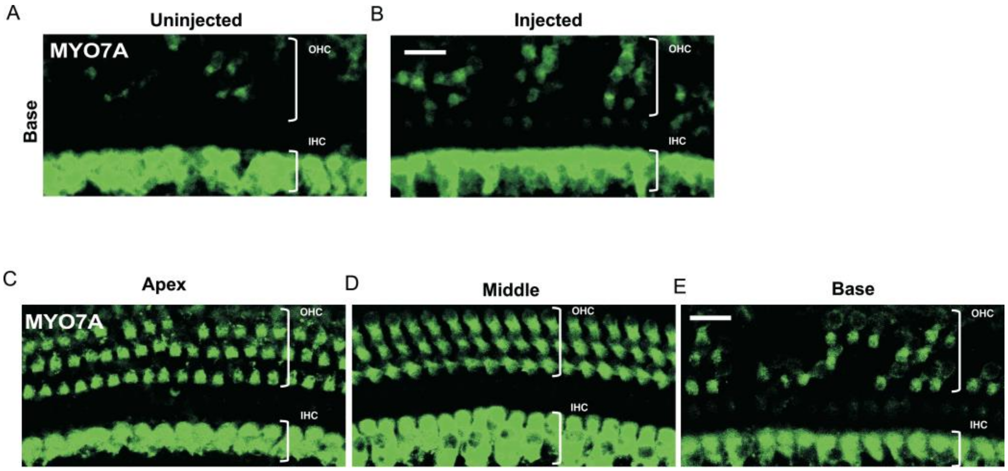

Fig. S8, AAV2-SaCas9-KKH-sgRNA-4 editing on hair cell survival. (A and B), Representative confocal z-stack images of whole mount cochleae basal turn from AAV2-SaCas9-KKH-sgRNA-4 uninjected **(A)** and injected **(B)** *Mir96*$^{14C>A/+}$ mice. Hair cells were stained for MYO7A (green). Scale bar, 20μm. **C-E,** Representative confocal z-stack images of whole mount cochleae from wild-type mice. Hair cells were stained for MYO7A (green). Scale bar, 20μm. Results were repeated independently in 3 cochleae.

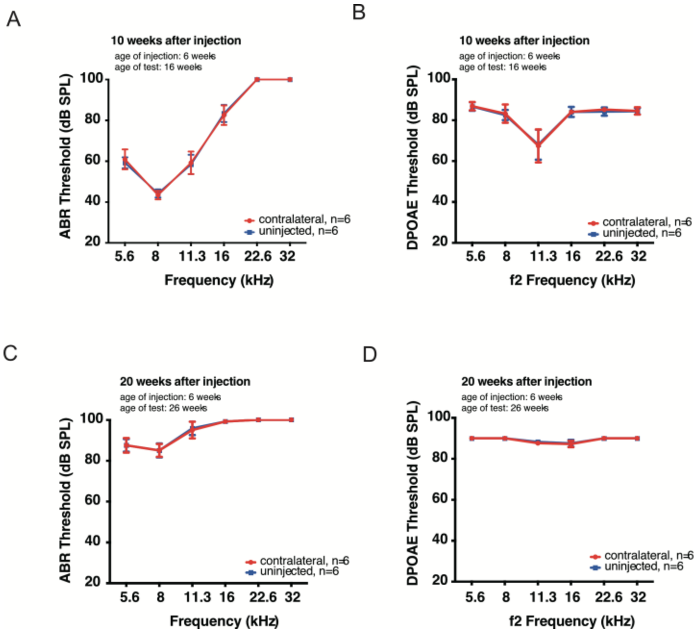

Fig. S9, The leakage expression of AAV2-SaCas9-KKH-sgRNA-4 in the contralateral uninjected ear did not enhance auditory function. (**A to D**), ABR **(A** and **C)** and DPOAE **(B** and **D)** thresholds of the uninjected contralateral ear from injected animals (red) and the ear from uninjected animals (blue) at 10 weeks **(A** and **B)** and 20 weeks **(C** and **D)** post-injection. Values and error bars reflect mean ± SEM. n=6.

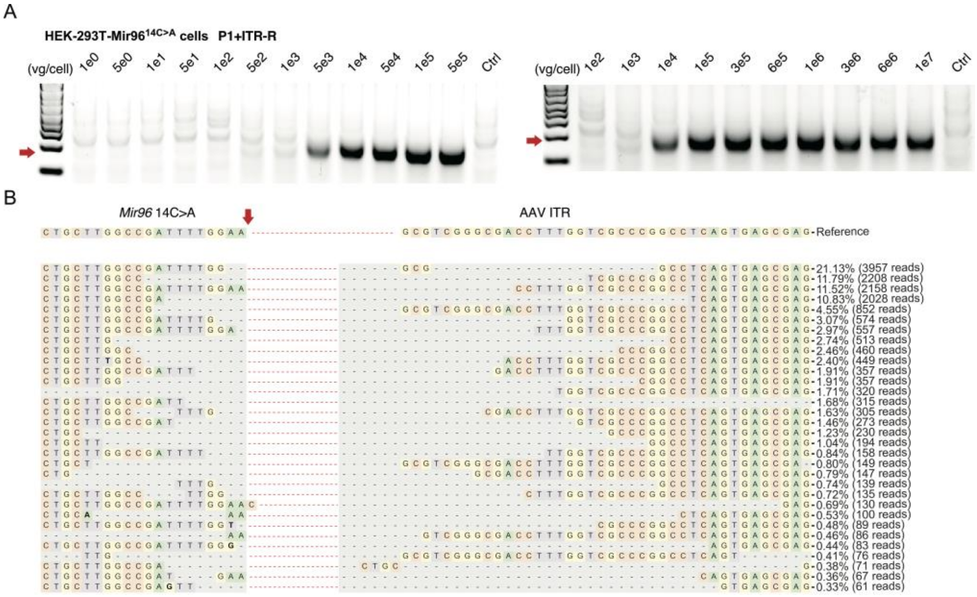

Fig. S10, Safety assessment of AAV delivery of editing complex in adult mice. (A), Gel image of the PCR showing the miR96-ITR integration fragment after different dosages of AAV treatment in HEK-miR96-14C>A cells. The horizontal red arrows indicate the location of integration amplicon. (**B)**, NGS results of miR96-ITR integration reads from AAV treated HEK-miR96-14C>A cells. The vertical red arrow indicates the double-stranded DNA cutting site.

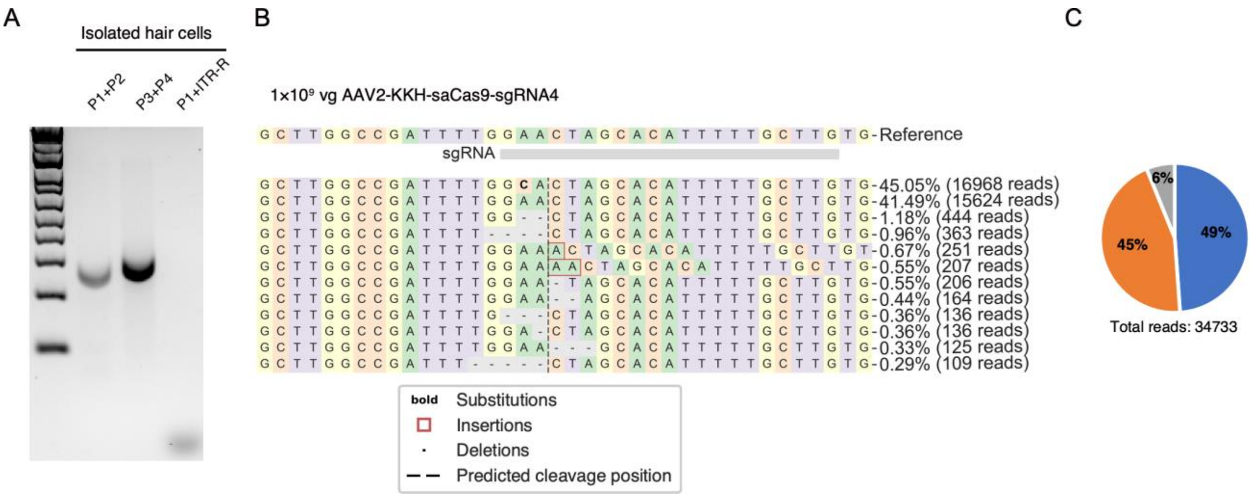

Fig. S11, Low titer AAV2 eliminated AAV vector integration. (A), Gel image of the PCR showing the SaCas9-KKH expression and miR96-ITR integration fragment after 1×10^9 vg AAV2 treatment. **(B)**, Representative NGS results of 1×10^9 vg AAV2-SaCas9-KKH-sgRNA-4 injected ears from *Mir96*$^{14C>A/+}$ mice. Reference sequence is the mutant allele. **(C)**, Pie chart showing the percentage of Mir96 wild-type allele reads (blue), 14C>A reads (orange), and indel-containing reads (grey) in the NGS results from panel B.

A

Name	Sequence	Mismatch	Genomic Location
miR-96 s1334	CAAGCAAAAATGTGCTAGTTC	0	miR-96
off-target-1	CAAGCAGAAATGTGCTAGGTC	2	intergenic:Gm19782-Fam135b
off-target-2	AAAGCAAAAGTGTGCTATTTC	3	intergenic:Tcf15-Csnk2a1
off-target-3	CATGCAAACATGAGCTAGTTC	3	intergenic:Fbxw7-1700036G14Rik
off-target-4	GAGGCAAAAATTTGCTAGTTC	3	intergenic:Gm20757-Nedd1
off-target-5	CAAGCAAAAATATTGTATTTC	4	intergenic:Hoxd1-Mtx2
off-target-6	CAAGAAAAACTGCTCTAGTTC	4	intergenic:Txn1-Txndc8
off-target-7	CAAACAAAAATGTACTATTTT	4	intergenic:Ptgfr-Gm31739
off-target-8	CAAGAAAAAATGTTCAAGATC	4	intergenic:Nfia-Tm2d1
off-target-9	CAAGCAAAAACTTGGTAGTTT	4	intergenic:Col11a1-Olfm3
off-target-10	GAAGCAGAAATGTGGTATTT	4	intron:Specc1

B

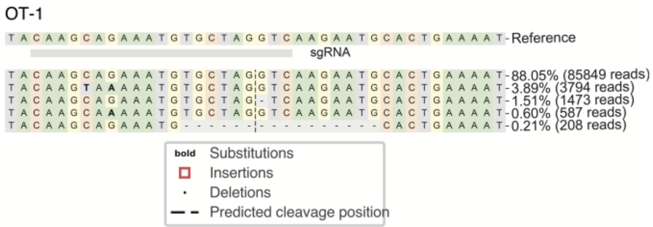

Fig. S12, off-target analysis. (A), The sequences of potential off-target genetic loci of SaCas9-KKH/sgRNA-4 in mouse genome. None of these loci were associated with hearing function. **(B),** The NGS result of off-target editing at the OT1 locus showed a low level of indel formation.

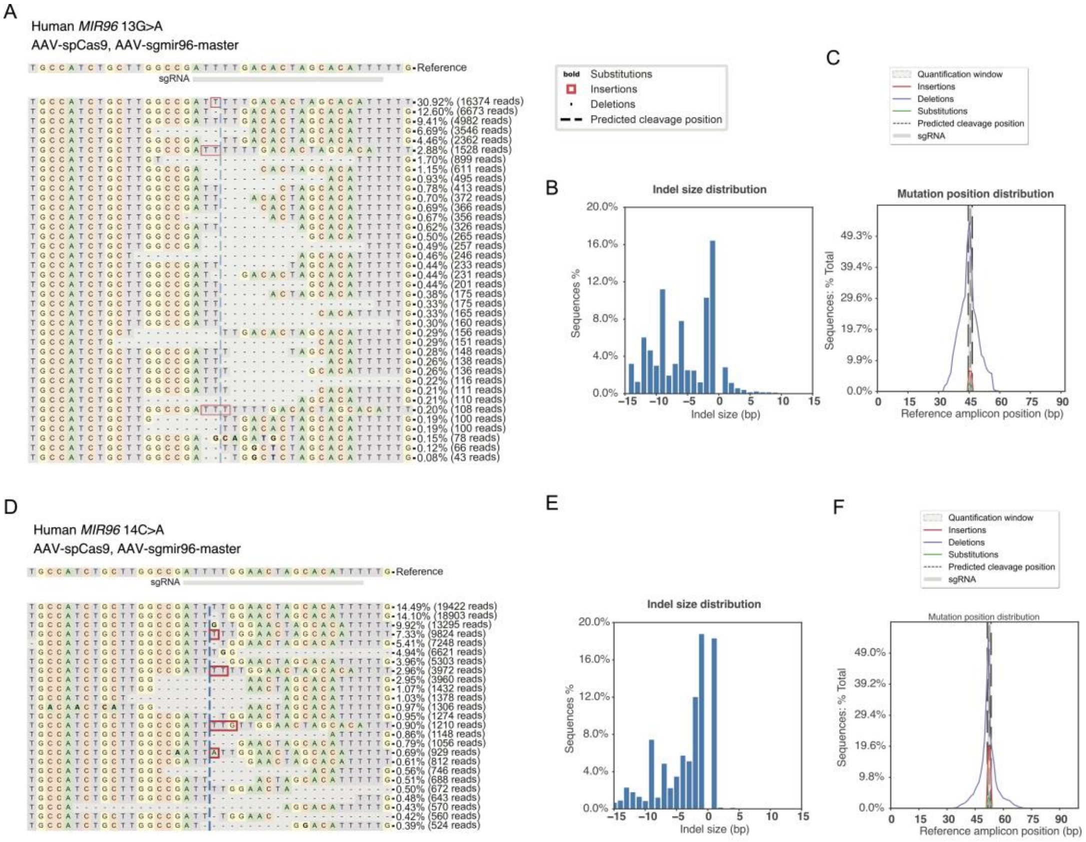

G

off-target-1	AAATGTAGAAATTCCAAAAT	4:intergenic:RAF1-TMEM40
off-target-2	AAATCTGTTTGCTCCAAAAT	4:exon:TSHZ2
off-target-3	ACATGTGTCAGATCCAAAAT	4:intergenic:RP11-396O20.2-RP11-222N13.1
off-target-4	AAATGTTCCTATTCCAAAAT	4:intron:NKAIN3
off-target-5	AAATGCCCCAGTTTCAAAAT	4:intron:TRAF3IP1
off-target-6	GAAAGAGCTAATTCCAAAAT	4:intergenic:KCND2-RNU6-581P
off-target-7	AAATGATCCTGTTCCAAAAT	4:intergenic:RNU6-793P-RP11-146I2.1
off-target-8	AAATGGGAAAATTCCAAAAT	4:intergenic:FOXI1-LINC01187
off-target-9	AAAAATGTAAGTTCCAAAAT	4:intergenic:CYP7B1-RP11-1D12.1
off-target-10	CAATGTGCCACCTCCAAAAT	4:intergenic:FAM46D-BRWD3

Fig. S13, Efficient genome editing targeting multiple *MIR96* seed region mutations in human cells. (A), Representative NGS results from spCas9/sgmiR96-master edited HEK-miR96 (13G to A) cells. **(B** and **C),** Indel profiles from spCas9/sgmiR96-master edited HEK-miR96 (13G to A) cells. Minus numbers represent deletions, plus numbers represent insertions. **(D),** Representative NGS results from spCas9/sgmiR96-master edited HEK-miR96 (14C to A) cells. **(E** and **F),** Indel profiles from spCas9/sgmiR96-master edited HEK-miR96 (14C to A) cells. Minus numbers represent deletions, plus numbers represent insertions. **(G),** The sequences of potential off-target genetic loci of spCas9/sgmiR96-master in the human genome. None of these loci were associated with hearing function.

AAV-CMV-SaCas9-KKH-sgRNA-4:

CCTGCAGGCAGCTGCGCGCTCGCTCGCTCACTGAGGCCGCCCGGGCGTCGGGCGACCTTTGGTCGCCCGGCCTCAGTGAGCGAGC
GAGCGCGCAGAGAGGGAGTGGCCAACTCCATCACTAGGGGTTCCTGCGGCCTCTAGACTCGAGGCGTTGACATTGATTATTGACT
AGTTATTAATAGTAATCAATTACGGGGTCATTAGTTCATAGCCCATATATGGAGTTCCGCGTTACATAACTTACGGTAAATGGCCC
GCCTGGCTGACCGCCCAACGACCCCCGCCCATTGACGTCAATAATGACGTATGTTCCCATAGTAACGCCAATAGGGACTTTCCATT
GACGTCAATGGGTGGAGTATTTACGGTAAACTGCCCACTTGGCAGTACATCAAGTGTATCATATGCCAAGTACGCCCCCTATTGAC
GTCAATGACGGTAAATGGCCCGCCTGGCATTATGCCCAGTACATGACCTTATGGGACTTTCCTACTTGGCAGTACATCTACGTATT
AGTCATCGCTATTACCATGGTGATGCGGTTTTGGCAGTACATCAATGGGCGTGGATAGCGGTTTGACTCACGGGGATTTCCAAGTC
TCCACCCCATTGACGTCAATGGGAGTTTGTTTTGGCACCAAAATCAACGGGACTTTCCAAAATGTCGTAACAACTCCGCCCCATTG
ACGCAAATGGGCGGTAGGCGTGTACGGTGGGAGGTCTATATAAGCAGAGCTCTCTGGCTAACTAGCCACCATGGGCAAACGCCC
AGCAGCTACAAAGAAGGCAGGTCAAGCCAAGAAAAAGAAAggaGCCCCAAAGAAGAAGCGGAAGGTCGGTtccAAGCGGAACTAC
ATCCTGGGCCTGGACATCGGCATCACCAGCGTGGGCTACGGCATCATCGACTACGAGACACGGGACGTGATCGATGCCGGCGTGC
GGCTGTTCAAAGAGGCCAACGTGGAAAACAACGAGGGCAGGCGGAGCAAGAGAGGCGCCAGAAGGCTGAAGCGGCGGAGGCGG
CATAGAATCCAGAGAGTGAAGAAGCTGCTGTTCGACTACAACCTGCTGACCGACCACAGCGAGCTGAGCGGCATCAACCCCTAC
GAGGCCAGAGTGAAGGGCCTGAGCCAGAAGCTGAGCGAGGAAGAGTTCTCTGCCGCCCTGCTGCACCTGGCCAAGAGAAGAGGC
GTGCACAACGTGAACGAGGTGGAAGAGGACACCGGCAACGAGCTGTCCACCAAAGAGCAGATCAGCCGGAACAGCAAGGCCCT
GGAAGAGAAATACGTGGCCGAACTGCAGCTGGAACGGCTGAAGAAAGACGGCGAAGTGCGGGGCAGCATCAACAGATTCAAGA
CCAGCGACTACGTGAAAGAAGCCAAACAGCTGCTGAAGGTGCAGAAGGCCTACCACCAGCTGGACCAGAGCTTCATCGACACCT
ACATCGACCTGCTGGAAACCCGGCGGACCTACTATGAGGGACCTGGCGAGGGCAGCCCCTTCGGCTGGAAGGACATCAAAGAAT
GGTACGAGATGCTGATGGGCCACTGCACCTACTTCCCCGAGGAACTGCGGAGCGTGAAGTACGCCTACAACGCCGACCTGTACAA
CGCCCTGAACGACCTGAACAATCTCGTGATCACCAGGGACGAGAACGAGAAGCTGGAATATTACGAGAAGTTCCAGATCATCGA
GAACGTGTTCAAGCAGAAGAAGAAGCCCACCCTGAAGCAGATCGCCAAAGAAATCCTCGTGAACGAAGAGGATATTAAGGGCTA
CAGAGTGACCAGCACCGGCAAGCCCGAGTTCACCAACCTGAAGGTGTACCACGACATCAAGGACATTACCGCCCGGAAAGAGAT
TATTGAGAACGCCGAGCTGCTGGATCAGATTGCCAAGATCCTGACCATCTACCAGAGCAGCGAGGACATCCAGGAAGAACTGAC
CAATCTGAACTCCGAGCTGACCCAGGAAGAGATCGAGCAGATCTCTAATCTGAAGGGCTATACCGGCACCCACAACCTGAGCCTG
AAGGCCATCAACCTGATCCTGGACGAGCTGTGGCACACCAACGACAACCAGATCGCTATCTTCAACCGGCTGAAGCTGGTGCCCA
AGAAGGTGGACCTGTCCCAGCAGAAAGAGATCCCCACCACCCTGGTGGACGACTTCATCCTGAGCCCCGTCGTGAAGAGAAGCTT
CATCCAGAGCATCAAAGTGATCAACGCCATCATCAAGAAGTACGGCCTGCCCAACGACATCATTATCGAGCTGGCCCGCGAGAA
GAACTCCAAGGACGCCCAGAAAATGATCAACGAGATGCAGAAGCGGAACCGGCAGACCAACGAGCGGATCGAGGAAATCATCC
GGACCACCGGCAAAGAGAACGCCAAGTACCTGATCGAGAAGATCAAGCTGCACGACATGCAGGAAGGCAAGTGCCTGTACAGCC
TGGAAGCCATCCCTCTGGAAGATCTGCTGAACAACCCCTTCAACTATGAGGTGGACCACATCATCCCCAGAAGCGTGTCCTTCGA
CAACAGCTTCAACAACAAGGTGCTCGTGAAGCAGGAAGAAAACAGCAAGAAGGGCAACCGGACCCCATTCCAGTACCTGAGCAG
CAGCGACAGCAAGATCAGCTACGAAACCTTCAAGAAGCACATCCTGAATCTGGCCAAGGGCAAGGGCAGAATCAGCAAGACCAA
GAAAGAGTATCTGCTGGAAGAACGGGACATCAACAGGTTCTCCGTGCAGAAAGACTTCATCAACCGGAACCTGGTGGATACCAG
ATACGCCACCAGAGGCCTGATGAACCTGCTGCGGAGCTACTTCAGAGTGAACAACCTGGACGTGAAAGTGAAGTCCATCAATGG
CGGCTTCACCAGCTTTCTGCGGCGGAAGTGGAAGTTTAAGAAAGAGCGGAACAAGGGGTACAAGCACCACGCCGAGGACGCCCT
GATCATTGCCAACGCCGATTTCATCTTCAAAGAGTGGAAGAAACTGGACAAGGCCAAAAAAGTGATGGAAAACCAGATGTTCGA
GGAAAAGCAGGCCGAGAGCATGcctgagatcgagacagagcaggaatacaaggaaattttcatcacccctcatcagattaaacacataaaggacttcaaagactataaatactctcatagggtg
gacaaaaaacccaatcgcaagctcattaatgacaccctgtactcaacacggaaggatgataaaggtaataccttgattgtgaataatcttaatggattgtatgacaaagataacgacaagctcaagaagctgatcaaca
agtctccagagaagctccttatgtatcaccacgacccacagacttatcagaaattgaaactgatcatggagcaatacggggatgagaagaacccactctacaaatattatgaggaaacaggtaattacctgaccaagta
ctccaagaaggataacggaccagtgatcaaaaagataaagtactatggcaacaaacttaatgcgcatttggacataactgacgattaccccaattctcgaaacaaggttgtgaagctctccctgaagccttatagatttg
acgtgtacctggataatggggtttataaattcgtcaccgtgaaaaatctggacgtgatcaaaaaggagaactattatgaagtaaactcaaagtgctatgaggaggcgaagaagctgaagaagatctccaatcaggccg
agttcatcgcttccttctataagaacgatctcatcaagatcaatggagagctttatcgcgtcattggtgtgaacaatgacttgctgaacaggatcgaagtcaatatgatagacattacctaccgggagtatctcgaaaacat
gaatgataaacggccgcctcacatcatcaagacaatcgcatctaaaactcagtcaataaaaaagtactctaccgatatcctggggaatctctatgaagtgaagtcaaagaagcacccacaaatcattaaaaaaggtA
AAAGGCCGGCGGCCACGAAAAAGGCCGGCCAGGCAAAAAAGAAAAAGGGATCCTACCCATACGATGTTCCAGATTACGCTTAAG
AATTCGCTGATCAGCCTCGACTGTGCCTTCTAGTTGCCAGCCATCTGTTGTTTGCCCCTCCCCCGTGCCTTCCTTGACCCTGGAAGG
TGCCACTCCCACTGTCCTTTCCTAATAAAATGAGGAAATTGCATCGCATTGTCTGAGTAGGTGTCATTCTATTCTGGGGGGTGGGG
TGGGGCAGGACAGCAAGGGGGAGGATTGGGAAGAGAATAGCAGGCATGCTGGGGAGGTACCGAGGGCCTATTTCCCATGATTCC
TTCATATTTGCATATACGATACAAGGCTGTTAGAGAGATAATTGGAATTAATTTGACTGTAAACACAAAGATATTAGTACAAAAT
ACGTGACGTAGAAAGTAATAATTTCTTGGGTAGTTTGCAGTTTTAAAATTATGTTTTAAAATGGACTATCATATGCTTACCGTAAC
TTGAAAGTATTTCGATTTCTTGGCTTTATATATCTTGTGGAAAGGACGAAACACCGAAGCAAAAATGTGCTAGTTCgttAtagtactctgtaa
tgaaaattacagaatctactaTaacaaggcaaaatgccgtgtttatctcgtcaacttgttggcgagaTTTTTTTGCGGCCGCAGGAACCCCTAGTGATGGAGTTGGCCACTC
CCTCTCTGCGCGCTCGCTCGCTCACTGAGGCCGGGCGACCAAAGGTCGCCCGACGCCCGGGCTTTGCCCGGGCGGCCTCAGTGAG
CGAGCGAGCGCGCAGCTGCCTGCAGG

ITR, CMV Promoter, NLSs, SaCas9-KKH coding sequence, HA Tag, PolyA, U6 Promoter, sgRNA

Fig. S14. DNA sequence of AAV-CMV-SaCas9-KKH-sgRNA-4.

AAV-U1A-spCas9-PA:

CCTGCAGGCAGCTGCGCGCTCGCTCGCTCACTGAGGCCGCCCGGGCGTCGGGCGACCTTTGGTCGCCCGGCCTCAGTGAGCGAGC
GAGCGCGCAGAGAGGGAGTGGCCAACTCCATCACTAGGGGTTCCTGCGGCCTCTAGAATGGAGGCGGTACTATGTAGATGAGAA
TTCAGGAGCAAACTGGAAAAGCAACTGCTTCCAAATATTTGTGATTTTTACAGTGTAGTTTTGGAAAAACTCTTAGCCTACCAAT
TCTTCTAAGTGTTTTAAAATGTGGGAGCCAGTACACATGAAGTTATAGAGTGTTTTAATGAGGCTTAAATATTTACCGTAACTATG
AAATGCTACGCATATCATGCTGTTCAGGCTCCGTGGCCACGCAACTCATACTACCGGtgCCACCATGGGCAAACGCCCAGCAGCTA
CAAAGAAGGCAGGTCAAGCCAAGAAAAAGAAAggaGCCCCAAAGAAGAAGCGGAAGGTCGGTGGAtccGACAAGAAGTACAGCAT
CGGCCTGGACATCGGCACCAACTCTGTGGGCTGGGCCGTGATCACCGACGAGTACAAGGTGCCCAGCAAGAAATTCAAGGTGCT
GGGCAACACCGACCGGCACAGCATCAAGAAGAACCTGATCGGAGCCCTGCTGTTCGACAGCGGCGAAACAGCCGAGGCCACCCG
GCTGAAGAGAACCGCCAGAAGAAGATACACCAGACGGAAGAACCGGATCTGCTATCTGCAAGAGATCTTCAGCAACGAGATGGC
CAAGGTGGACGACAGCTTCTTCCACAGACTGGAAGAGTCCTTCCTGGTGGAAGAGGATAAGAAGCACGAGCGGCACCCCATCTTC
GGCAACATCGTGGACGAGGTGGCCTACCACGAGAAGTACCCCACCATCTACCACCTGAGAAAGAAACTGGTGGACAGCACCGAC
AAGGCCGACCTGCGGCTGATCTATCTGGCCCTGGCCCACATGATCAAGTTCCGGGGCCACTTCCTGATCGAGGGCGACCTGAACC
CCGACAACAGCGACGTGGACAAGCTGTTCATCCAGCTGGTGCAGACCTACAACCAGCTGTTCGAGGAAAACCCCATCAACGCCA
GCGGCGTGGACGCCAAGGCCATCCTGTCTGCCAGACTGAGCAAGAGCAGACGGCTGGAAAATCTGATCGCCCAGCTGCCCGGCG
AGAAGAAGAATGGCCTGTTCGGAAACCTGATTGCCCTGAGCCTGGGCCTGACCCCCAACTTCAAGAGCAACTTCGACCTGGCCGA
GGATGCCAAACTGCAGCTGAGCAAGGACACCTACGACGACGACCTGGACAACCTGCTGGCCCAGATCGGCGACCAGTACGCCGA
CCTGTTTCTGGCCGCCAAGAACCTGTCCGACGCCATCCTGCTGAGCGACATCCTGAGAGTGAACACCGAGATCACCAAGGCCCCC
CTGAGCGCCTCTATGATCAAGAGATACGACGAGCACCACCAGGACCTGACCCTGCTGAAAGCTCTCGTGCGGCAGCAGCTGCCTG
AGAAGTACAAAGAGATTTTCTTCGACCAGAGCAAGAACGGCTACGCCGGCTACATTGACGGCGGAGCCAGCCAGGAAGAGTTCT
ACAAGTTCATCAAGCCCATCCTGGAAAAGATGGACGGCACCGAGGAACTGCTCGTGAAGCTGAACAGAGAGGACCTGCTGCGGA
AGCAGCGGACCTTCGACAACGGCAGCATCCCCCACCAGATCCACCTGGGAGAGCTGCACGCCATTCTGCGGCGGCAGGAAGATTT
TTACCCATTCCTGAAGGACAACCGGGAAAAGATCGAGAAGATCCTGACCTTCCGCATCCCCTACTACGTGGGCCCTCTGGCCAGG
GGAAACAGCAGATTCGCCTGGATGACCAGAAAGAGCGAGGAAACCATCACCCCCTGGAACTTCGAGGAAGTGGTGGACAAGGGC
GCTTCCGCCCAGAGCTTCATCGAGCGGATGACCAACTTCGATAAGAACCTGCCCAACGAGAAGGTGCTGCCCAAGCACAGCCTGC
TGTACGAGTACTTCACCGTGTATAACGAGCTGACCAAAGTGAAATACGTGACCGAGGGAATGAGAAAGCCCGCCTTCCTGAGCGG
CGAGCAGAAAAAGGCCATCGTGGACCTGCTGTTCAAGACCAACCGGAAAGTGACCGTGAAGCAGCTGAAAGAGGACTACTTCAA
GAAAATCGAGTGCTTCGACTCCGTGGAAATCTCCGGCGTGGAAGATCGGTTCAACGCCTCCCTGGGCACATACCACGATCTGCTG
AAAATTATCAAGGACAAGGACTTCCTGGACAATGAGGAAAACGAGGACATTCTGGAAGATATCGTGCTGACCCTGACACTGTTTG
AGGACAGAGAGATGATCGAGGAACGGCTGAAAACCTATGCCCACCTGTTCGACGACAAAGTGATGAAGCAGCTGAAGCGGCGGA
GATACACCGGCTGGGGCAGGCTGAGCCGGAAGCTGATCAACGGCATCCGGGACAAGCAGTCCGGCAAGACAATCCTGGATTTCC
TGAAGTCCGACGGCTTCGCCAACAGAAACTTCATGCAGCTGATCCACGACGACAGCCTGACCTTTAAAGAGGACATCCAGAAAGC
CCAGGTGTCCGGCCAGGGCGATAGCCTGCACGAGCACATTGCCAATCTGGCCGGCAGCCCCGCCATTAAGAAGGGCATCCTGCAG
ACAGTGAAGGTGGTGGACGAGCTCGTGAAAGTGATGGGCCGGCACAAGCCCGAGAACATCGTGATCGAAATGGCCAGAGAGAAC
CAGACCACCCAGAAGGGACAGAAGAACAGCCGCGAGAGAATGAAGCGGATCGAAGAGGGCATCAAAGAGCTGGGCAGCCAGAT
CCTGAAAGAACACCCCGTGGAAAACACCCAGCTGCAGAACGAGAAGCTGTACCTGTACTACCTGCAGAATGGGCGGGATATGTA
CGTGGACCAGGAACTGGACATCAACCGGCTGTCCGACTACGATGTGGACCATATCGTGCCTCAGAGCTTTCTGAAGGACGACTCC
ATCGACAACAAGGTGCTGACCAGAAGCGACAAGAACCGGGGCAAGAGCGACAACGTGCCCTCCGAAGAGGTCGTGAAGAAGAT
GAAGAACTACTGGCGGCAGCTGCTGAACGCCAAGCTGATTACCCAGAGAAAGTTCGACAATCTGACCAAGGCCGAGAGAGGCGG
CCTGAGCGAACTGGATAAGGCCGGCTTCATCAAGAGACAGCTGGTGGAAACCCGGCAGATCACAAAGCACGTGGCACAGATCCT
GGACTCCCGGATGAACACTAAGTACGACGAGAATGACAAGCTGATCCGGGAAGTGAAAGTGATCACCCTGAAGTCCAAGCTGGT
GTCCGATTTCCGGAAGGATTTCCAGTTTTACAAAGTGCGCGAGATCAACAACTACCACCACGCCCACGACGCCTACCTGAACGCC
GTCGTGGGAACCGCCCTGATCAAAAAGTACCCTAAGCTGGAAAGCGAGTTCGTGTACGGCGACTACAAGGTGTACGACGTGCGG
AAGATGATCGCCAAGAGCGAGCAGGAAATCGGCAAGGCTACCGCCAAGTACTTCTTCTACAGCAACATCATGAACTTTTTCAAGA
CCGAGATTACCCTGGCCAACGGCGAGATCCGGAAGCGGCCTCTGATCGAGACAAACGGCGAAACCGGGGAGATCGTGTGGGATA
AGGGCCGGGATTTTGCCACCGTGCGGAAAGTGCTGAGCATGCCCCAAGTGAATATCGTGAAAAAGACCGAGGTGCAGACAGGCG
GCTTCAGCAAAGAGTCTATCCTGCCCAAGAGGAACAGCGATAAGCTGATCGCCAGAAAGAAGGACTGGGACCCTAAGAAGTACG
GCGGCTTCGACAGCCCCACCGTGGCCTATTCTGTGCTGGTGGTGGCCAAAGTGGAAAAGGGCAAGTCCAAGAAACTGAAGAGTGT
GAAAGAGCTGCTGGGGATCACCATCATGGAAAGAAGCAGCTTCGAGAAGAATCCCATCGACTTTCTGGAAGCCAAGGGCTACAA
AGAAGTGAAAAAGGACCTGATCATCAAGCTGCCTAAGTACTCCCTGTTCGAGCTGGAAAACGGCCGGAAGAGAATGCTGGCCTCT
GCCGGCGAACTGCAGAAGGGAAACGAACTGGCCCTGCCCTCCAAATATGTGAACTTCCTGTACCTGGCCAGCCACTATGAGAAGC
TGAAGGGCTCCCCCGAGGATAATGAGCAGAAACAGCTGTTTGTGGAACAGCACAAGCACTACCTGGACGAGATCATCGAGCAGA
TCAGCGAGTTCTCCAAGAGAGTGATCCTGGCCGACGCTAATCTGGACAAAGTGCTGTCCGCCTACAACAAGCACCGGGATAAGCC
CATCAGAGAGCAGGCCGAGAATATCATCCACCTGTTTACCCTGACCAATCTGGGAGCCCCTGCCGCCTTCAAGTACTTTGACACC
ACCATCGACCGGAAGAGGTACACCAGCACCAAAGAGGTGCTGGACGCCACCCTGATCCACCAGAGCATCACCGGCCTGTACGAG
ACACGGATCGACCTGTCTCAGCTGGGAGGCGACAAAAGGCCGGCGGCCACGAAAAAGGCCGGCCAGGCAAAAAAGAAAAAGTG
AGGAtccAATAAAGGAAATTTATTTTCATTGCAATAGTGTGTTGGAATTTTTTGTGTCTCTCAGCAGGAACCCCTAGTGATGGAGTT
GGCCACTCCCTCTCTGCGCGCTCGCTCGCTCACTGAGGCCGGGCGACCAAAGGTCGCCCGACGCCCGGGCTTTGCCCGGGCGGCC
TCAGTGAGCGAGCGAGCGCGCAGCTGCCTGCAGG

ITR, U1A Promoter, NLSs, spCas9 coding sequence, PolyA

Fig. S15, DNA sequence of AAV-U1A-spCas9-PA.

AAV-sgmir96-Master:

CCTGCAGGCAGCTGCGCGCTCGCTCGCTCACTGAGGCCGCCCGGGCGTCGGGCGACCTTTGGTCGCCCGGCCTCAGTGAGCGAGC
GAGCGCGCAGAGAGGGAGTGGCCAACTCCATCACTAGGGGTTCCTGCGGCCGCACGCGTGAGGGCCTATTTCCCATGATTCCTTC
ATATTTGCATATACGATACAAGGCTGTTAGAGAGATAATTGGAATTAATTTGACTGTAAACACAAAGATATTAGTACAAAATACG
TGACGTAGAAAGTAATAATTTCTTGGGTAGTTTGCAGTTTTAAAATTATGTTTTAAAATGGACTATCATATGCTTACCGTAACTTG
AAAGTATTTCGATTTCTTGGCTTTATATATCTTGTGGAAAGGACGAAACACCGAATGTGCTAGTTCCAAAATGTTTCAGAGCTATG
CTGGAAACAGCATAGCAAGTTGAAATAAGGCTAGTCCGTTATCAACTTGAAAAAGTGGCACCGAGTCGGTGCTTTTTTTCTAGAG
GGTACCGGGGCCCGGTCGACTAGTTATTAATAGTAATCAATTACGGGGTCATTAGTTCATAGCCCATATATGGAGTTCCGCGTTAC
ATAACTTACGGTAAATGGCCCGCCTGGCTGACCGCCCAACGACCCCCGCCCATTGACGTCAATAATGACGTATGTTCCCATAGTA
ACGCCAATAGGGACTTTCCATTGACGTCAATGGGTGGAGTATTTACGGTAAACTGCCCACTTGGCAGTACATCAAGTGTATCATAT
GCCAAGTACGCCCCCTATTGACGTCAATGACGGTAAATGGCCCGCCTGGCATTATGCCCAGTACATGACCTTATGGGACTTTCCTA
CTTGGCAGTACATCTACGTATTAGTCATCGCTATTACCATGGTGATGCGGTTTTGGCAGTACATCAATGGGCGTGGATAGCGGTTT
GACTCACGGGGATTTCCAAGTCTCCACCCCATTGACGTCAATGGGAGTTTGTTTTGGCACCAAAATCAACGGGACTTTCCAAAATG
TCGTAACAACTCCGCCCCATTGACGCAAATGGGCGGTAGGCGTGTACGGTGGGAGGTCTATATAAGCAGAGCTGGTTTAGTGAAC
CGTCAGATCCGCTAGCGCTACCGGTCGCCACCATGGTGAGCAAGGGCGAGGAGCTGTTCACCGGGGTGGTGCCCATCCTGGTCGA
GCTGGACGGCGACGTAAACGGCCACAAGTTCAGCGTGTCCGGCGAGGGCGAGGGCGATGCCACCTACGGCAAGCTGACCCTGAA
GTTCATCTGCACCACCGGCAAGCTGCCCGTGCCCTGGCCCACCCTCGTGACCACCCTGACCTACGGCGTGCAGTGCTTCAGCCGCT
ACCCCGACCACATGAAGCAGCACGACTTCTTCAAGTCCGCCATGCCCGAAGGCTACGTCCAGGAGCGCACCATCTTCTTCAAGGA
CGACGGCAACTACAAGACCCGCGCCGAGGTGAAGTTCGAGGGCGACACCCTGGTGAACCGCATCGAGCTGAAGGGCATCGACTT
CAAGGAGGACGGCAACATCCTGGGGCACAAGCTGGAGTACAACTACAACAGCCACAACGTCTATATCATGGCCGACAAGCAGAA
GAACGGCATCAAGGTGAACTTCAAGATCCGCCACAACATCGAGGACGGCAGCGTGCAGCTCGCCGACCACTACCAGCAGAACAC
CCCCATCGGCGACGGCCCCGTGCTGCTGCCCGACAACCACTACCTGAGCACCCAGTCCGCCCTGAGCAAAGACCCCAACGAGAAG
CGCGATCACATGGTCCTGCTGGAGTTCGTGACCGCCGCCGGGATCACTCTCGGCATGGACGAGCTGTACAAGTCCGGACTCAGAT
CTCGATAACCTGCAGCGAATTCGATATCAAGCTTATCGATACCGAGCGCTGCTCGAGAGATCTACGGGTGGCATCCCTGTGACCC
CTCCCCAGTGCCTCTCCTGGCCCTGGAAGTTGCCACTCCAGTGCCCACCAGCCTTGTCCTAATAAAATTAAGTTGCATCATTTTGTC
TGACTAGGTGTCCTTCTATAATATTATGGGGTGGAGGGGGGTGGTATGGAGCAAGGGGCAAGTTGGGAAGACAACCTGTAGGGC
CTGCGGGGTCTATTGGGAACCAAGCTGGAGTGCAGTGGCACAATCTTGGCTCACTGCAATCTCCGCCTCCTGGGTTCAAGCGATTC
TCCTGCCTCAGCCTCCCGAGTTGTTGGGATTCCAGGCATGCATGACCAGGCTCAGCTAATTTTTGTTTTTTTGGTAGAGACGGGGTT
TCACCATATTGGCCAGGCTGGTCTCCAACTCCTAATCTCAGGTGATCTACCCACCTTGGCCTCCCAAATTGCTGGGATTACAGGCG
TGAACCACTGCTCCCTTCCCTGTCCTTCTGATTTTGTAGGTAACCACGCAGGAAGAGGGCCTATTTCCCATGATTCCTTCATATTTG
CATATACGATACAAGGCTGTTAGAGAGATAATTGGAATTAATTTGACTGTAAACACAAAGATATTAGTACAAAATACGTGACGTA
GAAAGTAATAATTTCTTGGGTAGTTTGCAGTTTTAAAATTATGTTTTAAAATGGACTATCATATGCTTACCGTAACTTGAAAGTATT
TCGATTTCTTGGCTTTATATATCTTGTGGAAAGGACGAAACACCGAATGTGCTAGTGTCAAAATGTTTCAGAGCTATGCTGGAAAC
AGCATAGCAAGTTGAAATAAGGCTAGTCCGTTATCAACTTGAAAAAGTGGCACCGAGTCGGTGCTTTTTTTCAGGAAACAGCTAT
GACACGCGTGAGGGCCTATTTCCCATGATTCCTTCATATTTGCATATACGATACAAGGCTGTTAGAGAGATAATTGGAATTAATTT
GACTGTAAACACAAAGATATTAGTACAAAATACGTGACGTAGAAAGTAATAATTTCTTGGGTAGTTTGCAGTTTTAAAATTATGTT
TTAAAATGGACTATCATATGCTTACCGTAACTTGAAAGTATTTCGATTTCTTGGCTTTATATATCTTGTGGAAAGGACGAAACACC
GAATGTGCTAGAGCCAAAATGTTTCAGAGCTATGCTGGAAACAGCATAGCAAGTTGAAATAAGGCTAGTCCGTTATCAACTTGAA
AAAGTGGCACCGAGTCGGTGCTTTTTTGTGCGGACCGAGCGGCCGCAGGAACCCCTAGTGATGGAGTTGGCCACTCCCTCTCTGC
GCGCTCGCTCGCTCACTGAGGCCGGGCGACCAAAGGTCGCCCGACGCCCGGGCTTTGCCCGGGCGGCCTCAGTGAGCGAGCGAG
CGCGCAGCTGCCTGCAGG

ITR, CMV Promoter, EGFP, PolyA, U6 Promoter, sg14C, sg13G, sg15A

Fig. S16, DNA sequence of AAV- sgmir96-Master.

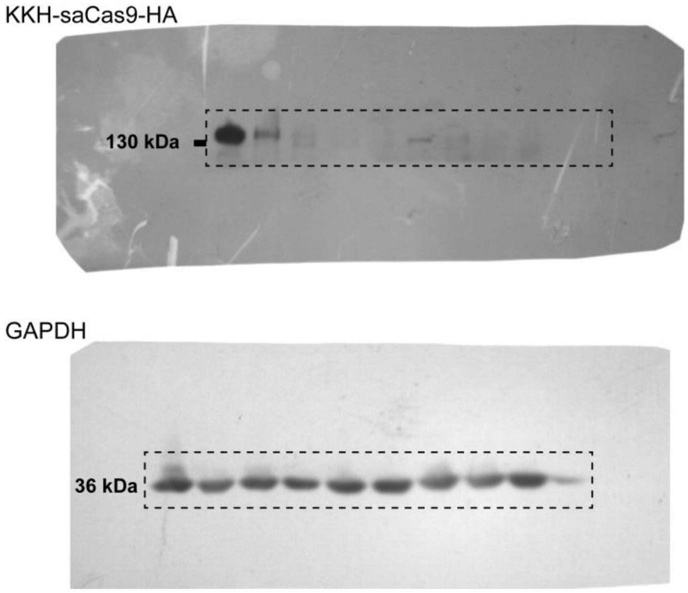

Fig. S17, Uncropped images of western blots.

Name	Sequence (5'-3')
rtmri96	CTCAACTGGTGTCGTGGAGTCGGCAATTCAGTTGAGCAAAAATGTG
qmiR96-F	TCGGCAGGTTTGGAACTAGCAC
qmiR96-R	CTCAACTGGTGTCGTGGA
P1	ACAGAGCAGAGACAGATCCGCGAG
P2	TGAGACACACAGACCCTGGGATG
P3	GCTTCAACAACAAGGTGCTCGTG
P4	CTGTTGATGTCCCGTTCTTCCAG
AAV ITR	GGAACCCCTAGTGATGGAGTT
mus-mir96-NGS-F	ACAGAGCAGAGACAGATCCGCGAG
mus-mir96-NGS-R	TGAGACACACAGACCCTGGGATG
hu-mir96-NGS-F	TCCGGAGCACCTTACCCACTTCTG
hu-mir96-NGS-R	GCTGGGGCCCTGACACAAGGATG

Table. S1, Primers used in this study.

Primer	Sequence (5'-3')	Primer	Sequence (5'-3')
hsp-OT1-F	CGGGATTTCACCATATTGGTCAGG	hsp-OT1-R	TGGTCTCGAACTCCTGACCTCAAG
hsp-OT2-F	AAGTGATGCATAAAACCTCGTGGG	hsp-OT2-R	TGAGGTTTCACCATGTTGGCCAGG
hsp-OT3-F	TGATTCCTAGGTCCTCTTTCACAG	hsp-OT3-R	GAGAGTGACAAGAAAGCTTCTATG
hsp-OT4-F	GTCCAAAGTCTCATCTGAGACAAG	hsp-OT4-R	AGCGTACCGTGGGTGTGAGACATG
hsp-OT5-F	AATAGCAGCAAACACCTAATAGGG	hsp-OT5-R	CATCAGGACTTTCAGATGACACAG
hsp-OT6-F	GCAAATAAATGATACACAAAGATG	hsp-OT6-R	TAAACATGATTGTCAATTTGGAGG
hsp-OT7-F	ACACAAAGACGACACGCCTGACAG	hsp-OT7-R	CATCTTAATCTGTTAGGCTGATGG
hsp-OT8-F	TTCAAAACAAAGGGTGTTTGGGGC	hsp-OT8-R	ATGTTCTTTTCCTGTGACCTCCTG
hsp-OT9-F	TACATTCACACACATACACACACG	hsp-OT10-F	ACACTGTAGAAACTCTGTTATTAG
hsp-OT10-F	ACACTGTAGAAACTCTGTTATTAG	hsp-OT10-R	GAGCCATAATATACTGAAGATCAG
msa-OT1-F	TGCACTGGGAAACTGAGCAAATAG	msa-OT1-R	TCTTGAATCCCAGTCACATCATTC
msa-OT2-F	GCTGGGATCTAAGTTTGATTGCTG	msa-OT2-R	AACACTAGGATGATGTTTAGAGGC
msa -OT3-F	AAGGTGGTCAGAACTTAGAGTTTG	msa -OT3-R	AAAGCTCATTTCAAACAAAGATCG
msa-OT4-F	TTTGGGCTCCTGCCATACTCTGTG	msa-OT4-R	GGAGAGTGGCCAGGAAAGCTAGTG
msa-OT5-F	AGTACCTAAAATATATACCCTAAG	msa-OT5-R	CAGTGGGTCCCCACATGCTCAGAG
msa-OT6-F	CTGACTTCATACTATGAATATGGG	msa-OT6-R	TTGCTACTGTTATGAATCGTAATG
msa-OT7-F	ATCTACAAAGCGTTGAACCTCGGG	msa-OT7-R	GATGTCTCTCTAAGATCCAGTGAG
msa-OT8-F	TCACTGTAGCCATTTCTCATGCAG	msa-OT8-R	CTGGTCTGTCTATAATTTAGTTGG
msa-OT9-F	ACTGGTACTAGAGCTATCTCTTCC	msa-OT9-R	ATGTAAGTGTATATATGTATATCTG
msa-OT10-F	CAGGCAGGCTGGCTGGAGCAGCAG	msa-OT10-R	CCTGAGCAATTATTAACTAGGCTG

Table. S2, Primers used for off-target analysis in this study.

Name	Sequence (5'-3')
sgRNA-1	AAATGTGCTAGTTCCAAAAT CGG
sgRNA-2	AAAATGTGCTAGTTCCAAAA TCG
sgRNA-3	AAAATGTGCTAGTTCCAAAA TCGG
sgRNA-4	CAAGCAAAAATGTGCTAGTTC CAAAAT
sgtdT-1	CAGACATGATAAGATACATTG ATGAGT
sgtdT-2	GTATGGCTGATTATGATCCTC TAGAGT
sg13	AAATGTGCTAGTGTCAAAAT CGG
sg15	AAATGTGCTAGAGCCAAAAT CGG

Table. S3, sgRNA protospacer sequences used in this study. Red fonts indicate the PAM sequence.
